# Taxonomy-Driven Core Scaffold Expansion Strategy for Comprehensive Profiling of Secondary and In Vivo Metabolites of Raphani Semen and Study on Promoting Gastrointestinal Motility

**DOI:** 10.3390/molecules31213942

**Published:** 2026-11-02

**Authors:** Xianming Lan, Ying Zhang, Hui Cao, Xintong Li, Weiting Liang, Jing Deng, Hao Qian, Menghua Wu, Zhiguo Ma

**Affiliations:** 1 College of Pharmacy, Jinan University, Guangzhou 511436, China; lanxianming596@163.com (X.L.);; 2 Research Center for Traditional Chinese Medicine of Lingnan (Southern China), Jinan University, Guangzhou 511436, China; 3 National Engineering Research Center for Modernization of Traditional Chinese Medicine Lingnan Resources Branch, Guangzhou 511436, China; 4 Guangdong Key Laboratory of Information of Traditional Chinese Medicine, Guangzhou 511436, China

**Keywords:** Taxonomy-Driven Core Scaffold Expansion Strategy, UHPLC-Q-Exactive Orbitrap MS/MS, Raphani Semen, metabolites, promoting gastrointestinal motility

## Abstract

Raphani Semen (RS) has two different application forms in the Chinese Pharmacopoeia (ChP): raw RS and stir-fried RS. However, the compounds of raw and stir-fried RS decoctions, in vivo metabolic pathways, and the effects on promoting gastrointestinal motility remain completely unclear. To address this, a Taxonomy-Driven Core Scaffold Expansion (TDCSE) strategy was developed to track compounds of raw and stir-fried RS decoctions and subsequent in vivo metabolic pathways. Then, a zebrafish model was used to evaluate the effect of raw and stir-fried RS decoctions on promoting gastrointestinal motility. Finally, through network pharmacology and molecular docking techniques, the potential targets and pathways of absorbed compounds were predicted. The results indicated that 26 compounds were detected in the stir-fried RS decoction. In addition to these 26 compounds, another 50 compounds were detected in the raw RS decoction. Moreover, a total of 123 metabolites (including prototypes) were detected after removing the duplications. A total of 18, 71, 59, and 4 metabolites were discovered in plasma, urine, feces, and liver tissue, respectively. Evaluation in a zebrafish model demonstrated that both raw RS and stir-fried RS decoctions possessed the effect of promoting gastrointestinal motility compared with the model group. The promoting gastrointestinal motility potential targets, PTGS2, TNF, PARP1, MTOR, etc., and the IL-17 pathway were predicted. Collectively, this study clarified the differences in compounds and in vivo metabolites of raw and stir-fried RS decoctions. It provided a reference for further investigation into the effect of promoting gastrointestinal motility of RS.

## 1. Introduction

Raphani Semen (RS), the dried seeds of *Raphanus sativus* L., is a traditional Chinese medicine [1]. The compounds of RS mainly include glucosinolates and sulfur-containing derivatives, phenylpropanoid derivatives, small organic acids and derivatives, flavone glycosides, terpenoids, steroids, oligosaccharides, etc. [2]. Focusing exclusively on glucosinolates and sulfur-containing derivatives and phenylpropanoid derivatives was significant. RS possesses different forms in the Chinese Pharmacopoeia (ChP): raw RS and stir-fried RS; raw RS is used less compared with stir-fried RS in clinical practice. Raw RS is used to vomit wind-phlegm. In addition, stir-fried RS is usually prepared by combining it with other herbs and taken as a decoction, such as the Sanzi Yangqing Decoction. Its traditional therapeutic functions include “directing qi downward to resolve phlegm” and “promoting digestion to eliminate stagnation”, and it is used for digestive disorders [3].

The differences in the compounds of raw and stir-fried RS were primarily attributable to the stir-frying process [4]. Studies have confirmed that stir-frying can inhibit the activity of endogenous enzymes in RS, thereby inhibiting the enzymatic hydrolysis of compounds [5]. At present, there have been preliminary studies on the compounds of RS before and after stir-frying. However, there is a lack of systematic research on the compounds in the decoctions of raw RS and stir-fried RS and their metabolic pathways in vivo after oral administration. Furthermore, the effects of raw RS and stir-fried RS decoctions on promoting gastrointestinal motility require further investigation.

To address the above issues, the Taxonomy-Driven Core Scaffold Expansion (TDCSE) strategy was developed to characterize the compounds of raw RS and stir-fried RS decoctions and track their metabolites in vivo. Conventional library searching offers high-throughput analysis and reliable identification of primary metabolites for which reference spectra were available. However, its application to complex natural products and metabolomics is substantially limited, primarily due to inadequate coverage of plant secondary metabolites and in vivo phase I and phase II metabolites. In recent years, Molecular Networking (MN) and Feature-Based Molecular Networking (FBMN) have been widely applied in the research and development of natural products [6,7]. MN constructs molecular networks solely based on cosine similarity of MS/MS spectra and neglects key information such as retention time and accurate mass, which may lead to misclassification of compounds. FBMN heavily depends on preprocessing steps such as feature extraction, alignment, and deconvolution. These steps are highly sensitive to parameter settings, which may introduce bias. These limitations are particularly pronounced in the absence of reliable reference databases, making it difficult to effectively identify and trace unknown compounds. TDCSE categorizes compounds by defining conserved biosynthetic core scaffolds as chemical “taxa” [8]. Compounds sharing a core scaffold undergo analogous fragmentation during tandem mass spectrometry and yield characteristic taxonomy-indicative product ions (TIPIs). By summarizing their characteristic TIPIs, we utilized these predefined TIPIs as customized filters to systematically screen raw LC-MS data, effectively rescuing metabolites previously discarded by FBMN. This enabled annotation of trace-level isomeric metabolites even when authentic reference spectra were absent from public databases. While MN provides a rapid spectral similarity overview and FBMN offers advanced quantitative analysis, TDCSE emphasizes progressive structural decoding and offers advantages in recursive validation and classification. TDCSE achieves lower false rates and higher structural specificity for targeted scaffold families in traditional Chinese medicine metabolite profiling.

In conventional mammalian models, limited optical accessibility can make real-time and continuous monitoring of intestinal peristalsis challenging. Therefore, the zebrafish (*Danio rerio*) model was employed as an alternative approach to assess gastrointestinal motility. The intestinal architecture, cellular composition, and motility regulatory networks of zebrafish exhibit high evolutionary conservation with mammals [9]. Moreover, the larvae permitted excellent optical transparency, enabling visualization of ingested chemical components [10]. This visualization-enabled in vivo approach may help address some limitations in real-time observation associated with conventional models, providing a useful platform for evaluating the gastrointestinal motility-promoting effects of raw and stir-fried RS decoctions.

Finally, network pharmacology and molecular docking frequently yielded biologically irrelevant false positives due to the untargeted screening of comprehensive databases [11,12,13]. In order to improve the accuracy of network pharmacology and molecular docking, compounds from RS decoctions that can be absorbed were selected to predict the potential targets and pathways of their effects on promoting gastrointestinal motility. Molecular docking was subsequently utilized to quantify the binding affinities between compounds and specific protein targets. However, it should be noted that network pharmacology and molecular docking only have the function of prediction. To determine the targets and pathways, a large number of experiments are required for verification.

This study systematically and rapidly clarified the compounds in raw RS and stir-fried RS decoctions and their metabolites in vivo by constructing the TDCSE strategy and combining it with the TIPIs technology. The zebrafish model was used to evaluate the promoting gastrointestinal motility of raw RS and stir-fried RS decoctions. The network pharmacology and molecular docking techniques were used to predict potential targets and pathways of RS decoctions that promote gastrointestinal motility. Generally, this study clarified the differences in compounds and in vivo metabolites of raw and stir-fried RS decoctions. It provided a reference for further investigation into the effect of promoting gastrointestinal motility of RS.

## 2. Results

### 2.1. The Construction of TDCSE Strategy

Due to the extremely complex compounds and in vivo metabolites of raw RS and stir-fried RS decoctions, it was necessary to develop an effective method for their analysis. Compounds derived from conserved biosynthetic pathways typically shared the same core scaffolds, which were defined herein as chemical “taxa”. During the mass spectrometry analysis, compounds possessing the same or similar core scaffolds exhibited analogous fragmentation patterns and yielded feature ions, which we termed TIPIs. These TIPIs can serve as structural signatures to further deduce the core scaffolds and substituents of related unknown compounds. This analytical approach facilitated the elucidation of compound structures, making it particularly advantageous for discovering novel compounds. Accordingly, to systematically and comprehensively characterize the glucosinolates and sulfur-containing derivatives, phenylpropanoid derivatives, and in vivo metabolites of raw RS and stir-fried RS decoctions, a novel TDCSE strategy was proposed [14,15].

Initially, the TIPIs of glucosinolates and phenylpropanoid derivatives of raw RS and stir-fried RS decoctions were summarized based on mass spectrometric fragmentation patterns. The structural variation among glucosinolates was determined exclusively by their side-chain substituents. Therefore, their TIPIs consisted of *m*/*z* 259, *m*/*z* 97, *m*/*z* 80, and *m*/*z* 76 + X, in negative mode. As for the majority of its sulfur-containing derivatives, they were typically generated in raw RS decoction. They yielded side-chain-substituted isothiocyanates or nitriles, and the TIPIs at *m*/*z* 60 + X were detected in positive mode. Here, X represents the mass of side-chain substituents. Additionally, phenylpropanoid derivatives primarily contained hydroxyl, methoxy, and glucose (Glc), leading to characteristic neutral losses of H_2_O (18 Da), CH_3_ (15 Da), CH_2_O (30 Da), CO_2_ (44 Da), Glc (162 Da), and their combinations. Consequently, the TIPIs at [M-H-nH_2_O]^−^, [M-H-nCH_3_]^−^, [M-H-CO_2_]^−^, [M-H-Glc]^−^, [M-H-nH_2_O-nCH_3_]^−^, [M-H-CO_2_-nH_2_O-nCH_3_]^−^, [M-H-Glc-nH_2_O-nCH_3_]^−^ and [M-H-Glc-CO_2_-nH_2_O-nCH_3_]^−^ were included.

Utilizing TIPIs as data filters, the raw LC-MS/MS datasets were systematically screened to identify compounds sharing specific biosynthetic pathways. These isolated candidate compounds shared a conserved core scaffold, but exhibited variant substituents. Then, compounds with the same TIPIs were classified into the same category. Subsequently, these classified compounds were verified. Firstly, we summarized their exact mass, isotope pattern, fragmentation behavior, and molecular formula. Then, the information on the compounds was compared with that of the standards. The accuracy of the previous classification of compounds was further verified, and compounds were preliminarily identified. Meanwhile, some relevant references and databases were utilized to assist in the identification of compounds. Ultimately, all the compounds in raw RS and stir-fried RS decoctions were characterized.

**Figure 1:**
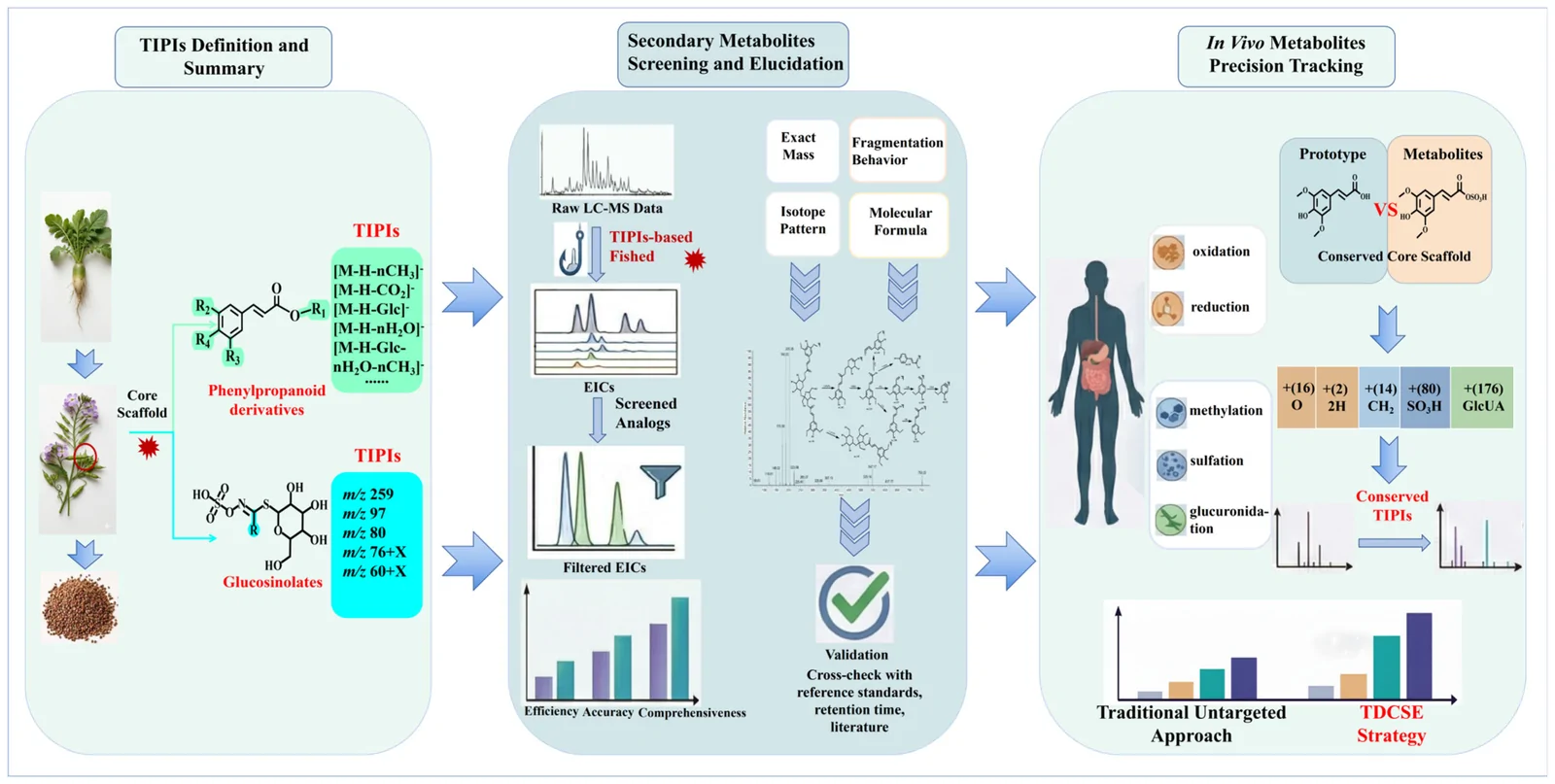
The summary diagram of the TDCSE strategy and methodology.

Crucially, the utility of the TDCSE strategy extended beyond the characterization of compounds in raw RS and stir-fried RS decoctions to the precise tracking of in vivo metabolites. Following oral administration, some RS compounds may undergo biotransformation through phase I reactions (e.g., oxidation, reduction, and demethylation) and phase II conjugation reactions (e.g., glucuronidation and sulfation). Despite these extensive structural modifications, the majority of metabolites preserved the core scaffold of prototypes. Consequently, they exhibited highly conserved fragmentation behaviors during tandem mass spectrometry and produced consistent TIPIs. Analytically, the precursor ions of these metabolites manifested predictable mass shifts (Δ *m*/*z*) relative to their prototypes, such as the addition of a methyl group (14 Da), oxygen (16 Da), a sulfate group (80 Da), a glucosyl moiety (162 Da), or a glucuronosyl moiety (176 Da). By utilizing the conserved TIPIs, these specific precursor mass shifts were systematically filtered. Finally, the TDCSE strategy was employed to conduct an efficient and comprehensive characterization of the complex in vivo metabolites (Figure 1).

**Figure 2:**
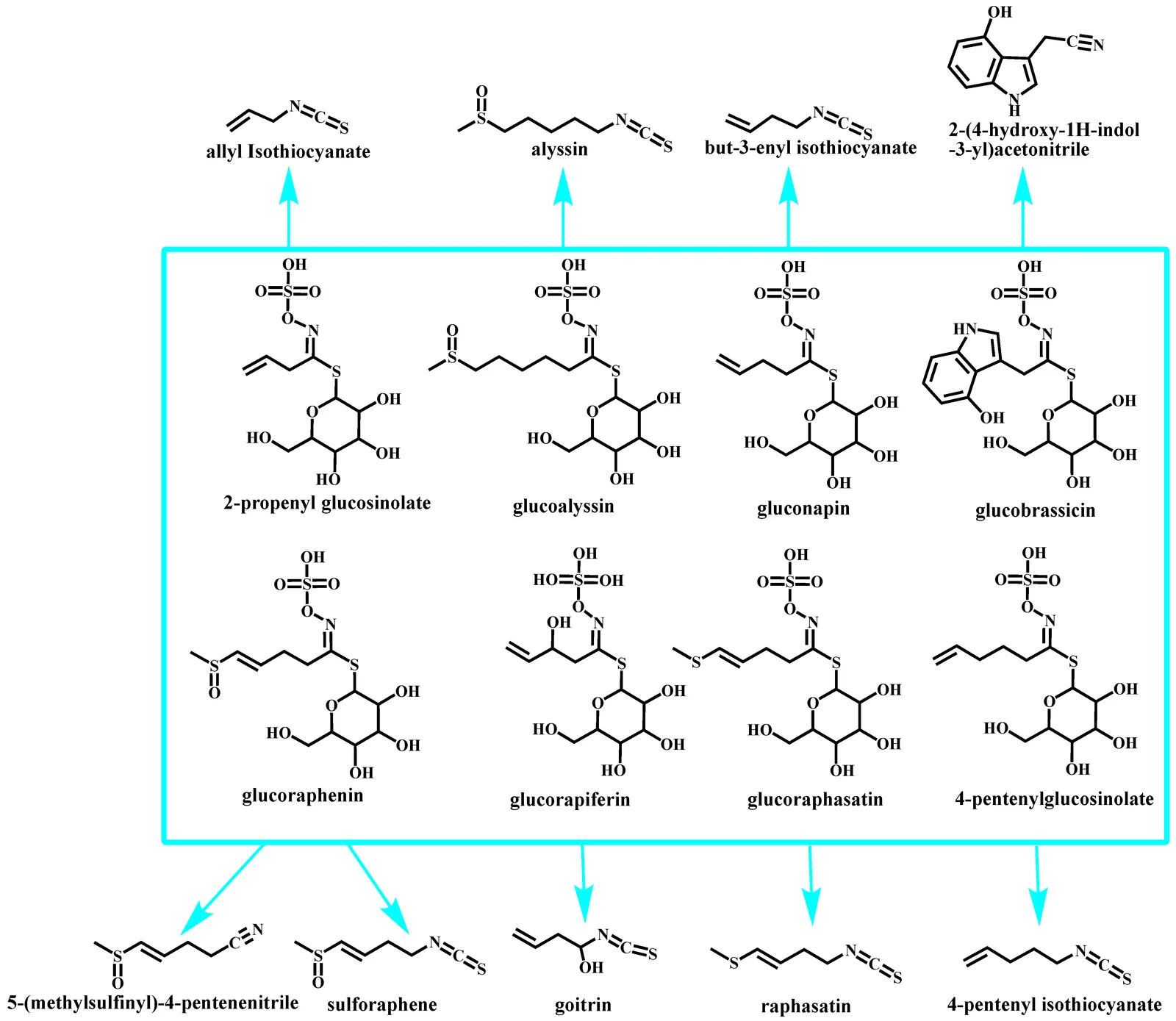
Possible transformations of glucosinolates in raw and stir-fried RS decoctions. (All the compounds were detected in the raw RS decoction, and the stir-fried RS decoction only detected the compounds within the blue rectangle).

**Figure 3:**
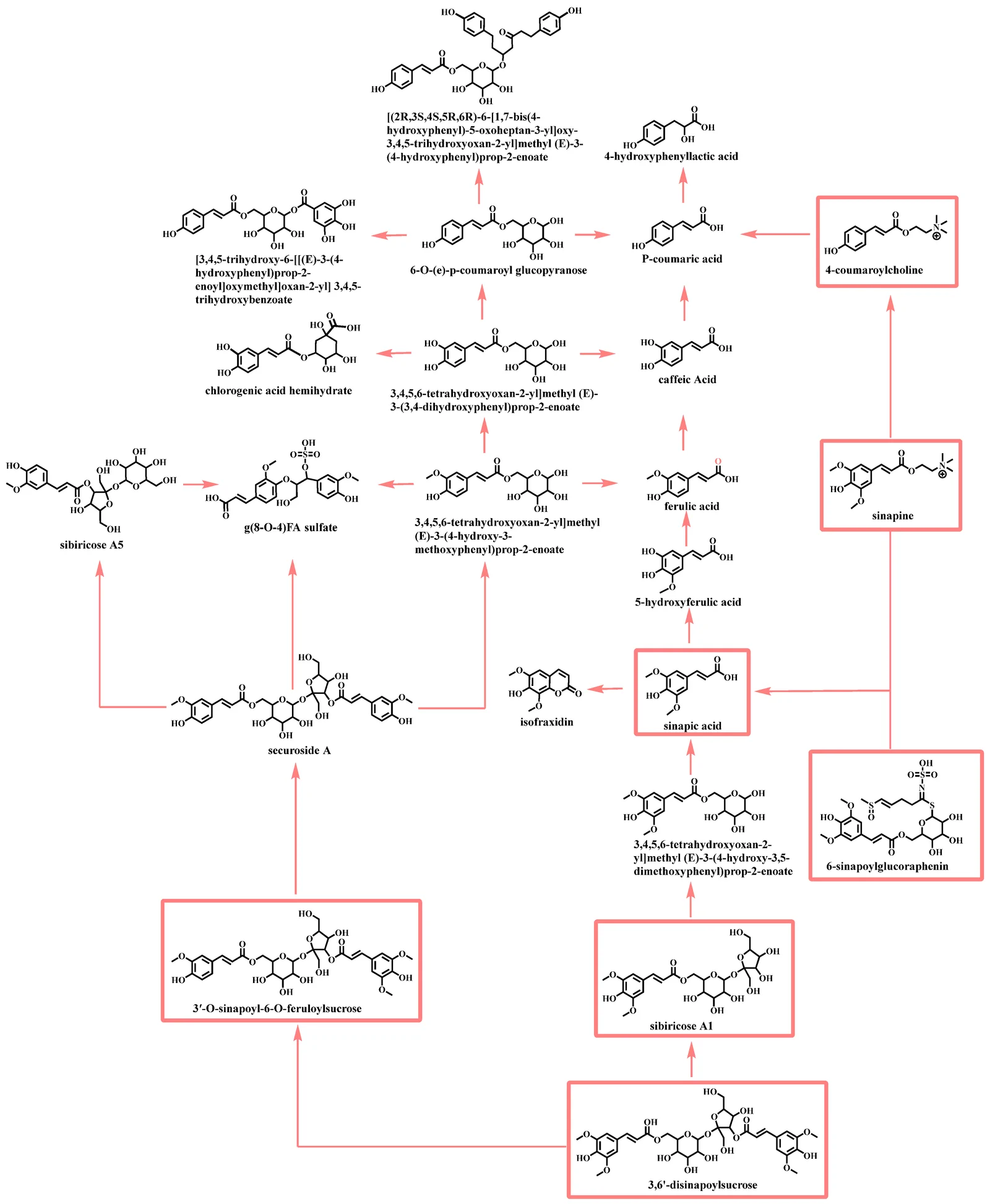
Possible transformations of phenylpropanoid derivatives in raw and stir-fried RS decoctions (all the compounds were detected in the raw RS decoction, whereas the stir-fried RS decoction only detected the compounds within the red rectangle).

Applying the TDCSE strategy, 26 compounds were detected in stir-fried RS decoction. In addition to these 26 compounds, another 50 compounds were detected in raw RS decoction. This might be related to the process of stir-frying to deactivate endogenous enzymes and prevent the decomposition of compounds during the water decoction process (Figure 2 and Figure 3, Table S1). The base peak chromatograms of the raw and stir-fried RS decoctions are shown in Figure S1.

### 2.2. Compounds Elucidation of Raw RS and Stir-Fried RS Decoctions

#### 2.2.1. Glucosinolates and Sulfur-Containing Derivatives

**Figure 4:**
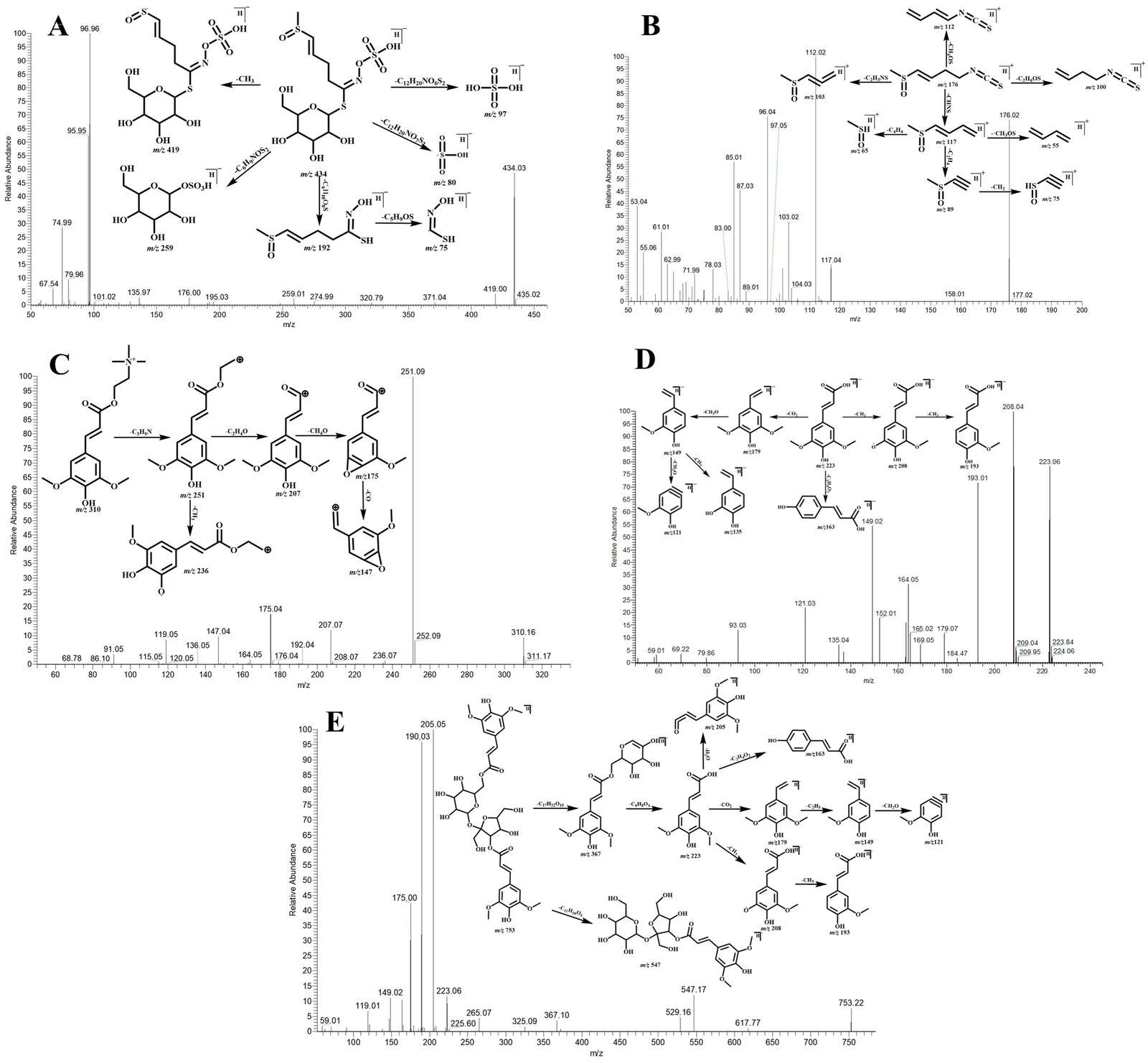
The mass spectrometric fragmentation patterns of glucoraphenin (**A**), sulforaphene (**B**), sinapine (**C**), sinapic acid (**D**), and 3,6′-disinapoylsucrose (**E**).

While glucosinolates can be detected in both positive and negative ion modes, their aglycones can only be detected in the positive ion mode. A1 produced an [M-H]^−^ ion at *m*/*z* 434.02548 with a retention time of 1.00 min (C_12_H_21_NO_10_S_3_, mass error of −0.14 ppm). It afforded the TIPIs at *m*/*z* 434 [M-H]^−^, *m*/*z* 419 [M-H-CH_3_]^−^, *m*/*z* 259 [M-H-C_6_H_9_NOS_2_]^−^, *m*/*z* 192 [M-H-C_6_H_10_O_8_S]^−^, *m*/*z* 97 [M-H-C_12_H_20_NO_6_S_2_]^−^, *m*/*z* 80 [M-H-C_12_H_20_NO_7_S_2_]^−^, and *m*/*z* 75 [M-H-C_6_H_10_O_8_S-C_5_H_9_OS]^−^. Furthermore, it displayed TIPIs at *m*/*z* 176 [M+H- C_6_H_12_O_9_S]^+^ in positive ion mode. Consequently, A1 was accurately identified as glucoraphenin compared with the reference (Figure 4A). A53 was eluted at 11.92 min and showed the molecular ion at *m*/*z* 176.01983. It was observed that 260 Da less than that of glucoraphenin suggested the absence of glucosyl and sulfate. In addition, it produced the TIPIs at *m*/*z* 117 [M+H-CHNS]^+^, *m*/*z* 117 [M+H-CH_4_OS]^+^, *m*/*z* 103 [M+H-C_2_H_3_NS]^+^, *m*/*z* 100 [M+H-C_2_H_6_OS]^+^, *m*/*z* 89 [M+H-CH_4_OS-C_2_H_4_]^+^, *m*/*z* 75 [M+H-CH_4_OS-C_2_H_4_-CH_2_]^+^, *m*/*z* 65 [M+H-CH_4_OS-C_4_H_4_]^+^, and *m*/*z* 55 [M+H-CH_4_OS-CH_3_OS]^+^. Finally, A53 was identified as sulforaphene (Figure 4B).

A16 was found at 5.23 min and showed an [M-H]^−^ ion at *m*/*z* 358.02719 (C_10_H_17_NO_9_S_2_). The DPIs at *m*/*z* 278 [M-H-SO_3_H]^−^, *m*/*z* 163 [M-H-C_4_H_6_NO_4_S]^−^, *m*/*z* 115 [M-H-C_6_H_10_O_8_S]^−^, and *m*/*z* 97 [M-H-C_10_H_16_NO_5_S]^−^ were observed. Then, it was speculated as 2-Propenyl glucosinolate or its isomer. Similarly, the molecular weight of A54 was 260 Da less than that of A16. Therefore, A54 was considered as allyl isothiocyanate or its isomer, which was the aglycone of A16.

A3 exhibited characteristic [M-H]^−^ ions at *m*/*z* 450.05678, with a retention time of 1.17 min. In their ESI-MS^2^ spectra, the TIPIs at *m*/*z* 386 [M-H-CH_3_OS]^−^, *m*/*z* 192 [M-H- C_6_H_10_O_9_S]^−^, *m*/*z* 97 [M-H-C_13_H_24_NO_6_S_2_]^−^, *m*/*z* 80 [M-H-C_13_H_24_NO_7_S_2_]^−^, and *m*/*z* 75 [M-H-C_12_H_23_O_9_S_2_]^−^ were generated. Thus, A3 was preliminarily deduced as glucoalyssin or its isomer. Correspondingly, A69 was tentatively speculated as the aglycone of A3 based on the 260 Da difference in their molecular masses.

In negative ion mode, A5 produced an [M-H]^−^ ion at *m*/*z* 372.04284 (C_11_H_19_NO_9_S_2_) with a mass error of −0.07 ppm. In its ESI-MS^2^ spectra, the TIPIs were assigned as *m*/*z* 97 [M-H-C_11_H_18_NO_5_S]^−^, *m*/*z* 80 [M-H-C_11_H_18_NO_6_S]^−^, and *m*/*z* 75 [M-H-C_10_H_17_O_8_S_2_]^−^. Hence, A5 was predicted as gluconapin. A58 was observed 260 Da less than that of gluconapin, which was deduced as but-3-enyl isothiocyanate or its isomer.

A14, with a retention time of 5.06 min, exhibited an [M-H]^−^ ion at *m*/*z* 418.03056. The ESI-MS/MS fragmentation patterns revealed the TIPIs at *m*/*z* 195 [M-H-C_6_H_10_NO_4_S_2_]^−^, *m*/*z* 176 [M-H-C_6_H_10_O_8_S]^−^, *m*/*z* 97 [M-H-C_12_H_20_NO_5_S_2_]^−^, *m*/*z* 80 [M-H-C_12_H_20_NO_6_S_2_]^−^, and *m*/*z* 80 [M-H-C_11_H_19_O_8_S_2_]^−^. Based on its fragmentation behavior and structural features, A14 was tentatively presumed as glucoraphasatin or its isomer. Likewise, the molecular weight of A64 was 260 Da less than that of A14. Therefore, A64 was considered as raphasatin or its isomer, which was the aglycone of A14.

#### 2.2.2. Phenylpropanoid Derivatives

A36 exhibited characteristic [M+H]^+^ ions at *m*/*z* 310.16489, with a retention time of 10.22 min. In its ESI-MS^2^ spectra, the TIPIs at *m*/*z* 251 [M+H-C_3_H_9_N]^+^, *m*/*z* 236 [M+H-C_3_H_9_N-CH_3_]^+^, *m*/*z* 207 [M+H-C_3_H_9_N-C_2_H_4_O]^+^, *m*/*z* 175 [M+H-C_3_H_9_N-C_2_H_4_O-CH_4_O]^+^, *m*/*z* 147 [M+H-C_3_H_9_N-C_2_H_4_O-CH_4_O-CO]^+^, and *m*/*z* 115 [M+H-C_3_H_9_N-C_2_H_4_O-CH_4_O-C_2_H_4_O_2_]^+^ were generated. Thus, it was unambiguously characterized as sinapine compared with the reference standard (Figure 4C).

A62 yielded [M-H]^−^ ions at *m*/*z* 223.06119 (C_11_H_12_O_5_) with a retention time of 13.67 min. Additionally, it showed the TIPIs at *m*/*z* 208 [M-H-CH_3_]^−^, *m*/*z* 193 [M-H-2CH_3_]^−^, *m*/*z* 163 [M-H-C_2_H_4_O_2_]^−^, *m*/*z* 179 [M-H-CO_2_]^−^, *m*/*z* 149 [M-H-CO_2_-C_2_H_5_]^−^, *m*/*z* 135 [M-H-CO_2_-C_2_H_5_-CH_2_]^−^, and *m*/*z* 121 [M-H-CO_2_-C_2_H_5_-C_2_H_4_]^−^. Consequently, compared with the reference standard, A62 was definitely identified as sinapic acid (Figure 4D).

A69 was eluted at 16.31 min and showed the deprotonated molecular ion at *m*/*z* 753.22475. It was 532 Da (223 + 162 + 142 Da) more than that of sinapic acid, which indicated the presence of glucosyl and sinapic acid. In addition, it produced the TIPIs at *m*/*z* 547 [M-H-C_11_H_10_O_4_]^−^, *m*/*z* 367 [M-H-C_17_H_22_O_10_]^−^, *m*/*z* 223 [M-H-C_17_H_22_O_10_-C_6_H_8_O_4_]^−^, and *m*/*z* 205 [M-H-C_17_H_22_O_10_-C_6_H_8_O_4_-H_2_O]^−^. Consequently, A69 was identified as 3,6′-disinapoylsucrose compared with the reference standard (Figure 4E).

Three isomeric compounds, A32, A45, and A48, exhibited [M-H]^−^ ions at *m*/*z* 547.16684, corresponding to a mass increment of 324 Da (2 × 162 Da) relative to sinapic acid. Moreover, the TIPIs at *m*/*z* 367 [M-H-C_6_H_12_O_6_]^−^, *m*/*z* 325 [M-H-C_8_H_14_O_7_]^−^, *m*/*z* 295 [M-H-C_9_H_18_O_8_]^−^, *m*/*z* 265 [M-H-C_9_H_18_O_8_-2CH_3_]^−^, *m*/*z* 223 [M-H-2C_6_H_12_O_6_]^−^, *m*/*z* 205 [M-H-2C_6_H_12_O_6_-H_2_O]^−^, and *m*/*z* 190 [M-H-2C_6_H_12_O_6_-H_2_O-CH_3_]^−^ were all observed. Therefore, A48 was identified as sibiricose A1 compared with the reference standard; A32 and A45 were deduced as sibiricose A1 isomers.

### 2.3. In Vivo Metabolites of Raw RS and Stir-Fried RS Decoctions

**Figure 5:**
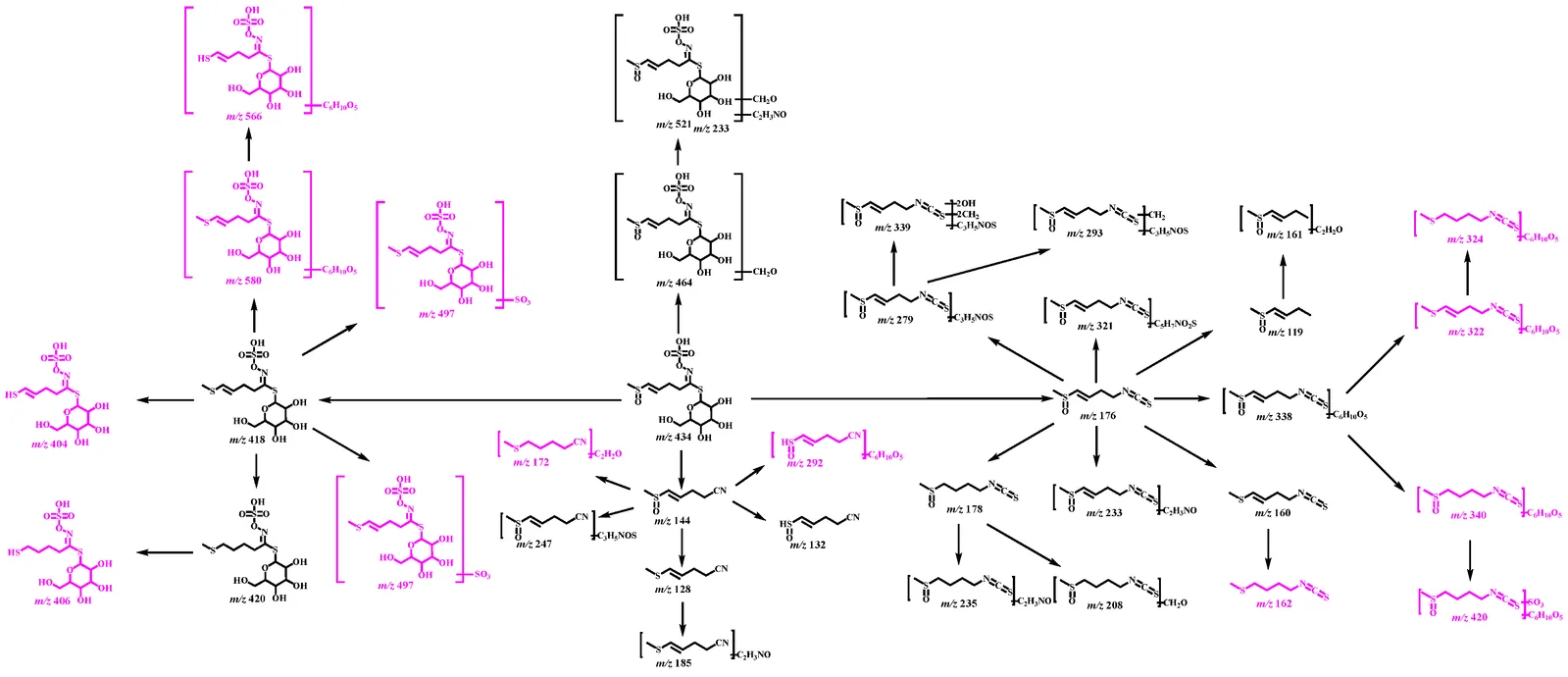
The proposed metabolic pathways of glucosinolates and their sulfur-containing derivatives of raw RS and stir-fried RS decoctions in vivo (metabolites in purple were only detected in stir-fried RS decoction).

**Figure 6:**
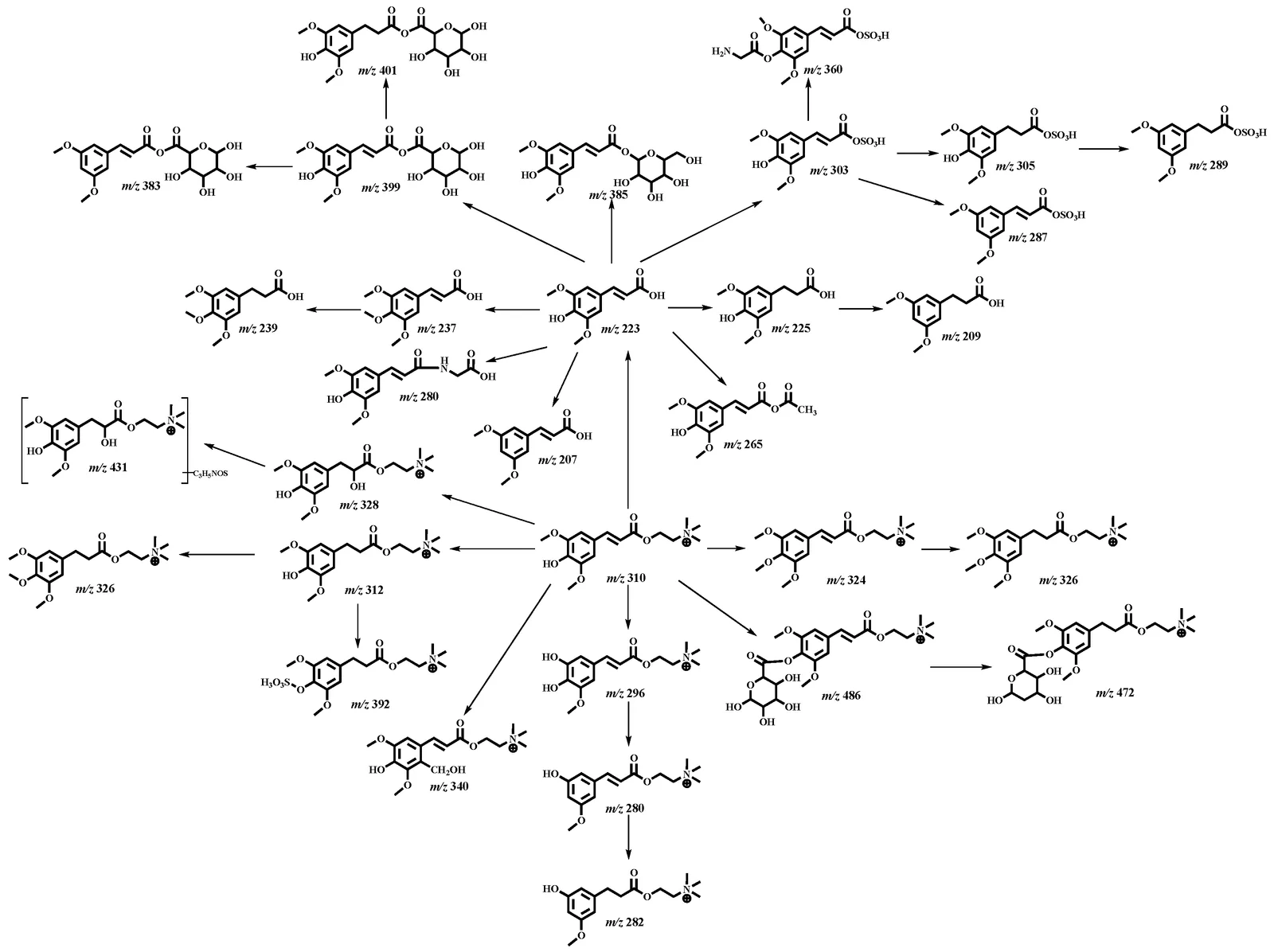
The proposed metabolic pathways of phenylpropanoid derivatives in raw RS and stir-fried RS decoctions in vivo.

To elucidate the systemic exposure and metabolic pathways of raw RS and stir-fried RS decoctions following oral administration, diverse biological matrices were systematically analyzed utilizing UHPLC-Q-Exactive Orbitrap MS/MS. A total of 123 (including prototypes) metabolites were detected after removing the duplications. In total, 18, 71, 59, and 4 metabolites were discovered in plasma, urine, feces, and liver tissue, respectively (Figure 5 and Figure 6, Table S2). The metabolites detected in plasma provided a basis for further investigation into their potential roles in promoting gastrointestinal motility. The base peak chromatograms of plasma, urine, feces, and liver tissue are shown in Figures S2, S3, S4, and S5, respectively.

M123 was eluted at 19.20 min and exhibited its [M-H]^−^ ion at *m*/*z* 464.03604, with a speculated molecular formula of C_13_H_23_NO_11_S_3_. Compared with glucoraphenin, the mass of M123 had increased by 30 Da, which indicated that glucoraphenin may have undergone hydroxymethylation to form M123. In its ESI-MS/MS spectrum, the TIPIs such as *m*/*z* 318 [M-H-Glc]^−^, *m*/*z* 206 [M-H-C_6_H_12_O_9_S]^−^, *m*/*z* 97 [M-H-C_13_H_22_NO_7_S_2_]^−^, and *m*/*z* 80 [M-H-C_13_H_22_NO_8_S_2_]^−^ were also observed. Based on these observations, M123 was tentatively speculated as an isomer of hydroxymethylation glucoraphenin.

With a retention time of 1.59 min, M8 displayed an [M-H]^−^ ion at *m*/*z* 404.01491 (C_11_H_19_NO_9_S_3_). Additionally, its mass weight was 14 Da less than that of glucoraphasatin, and the TIPIs at *m*/*z* 97 [M-H-C_11_H_18_NO_5_S_2_]^−^ and *m*/*z* 80 [M-H-C_11_H_18_NO_6_S_2_]^−^ were all observed, indicating that a methylation reaction might occur. Consequently, M8 was speculated as an isomer of demethylation glucoraphasatin.

**Figure 7:**
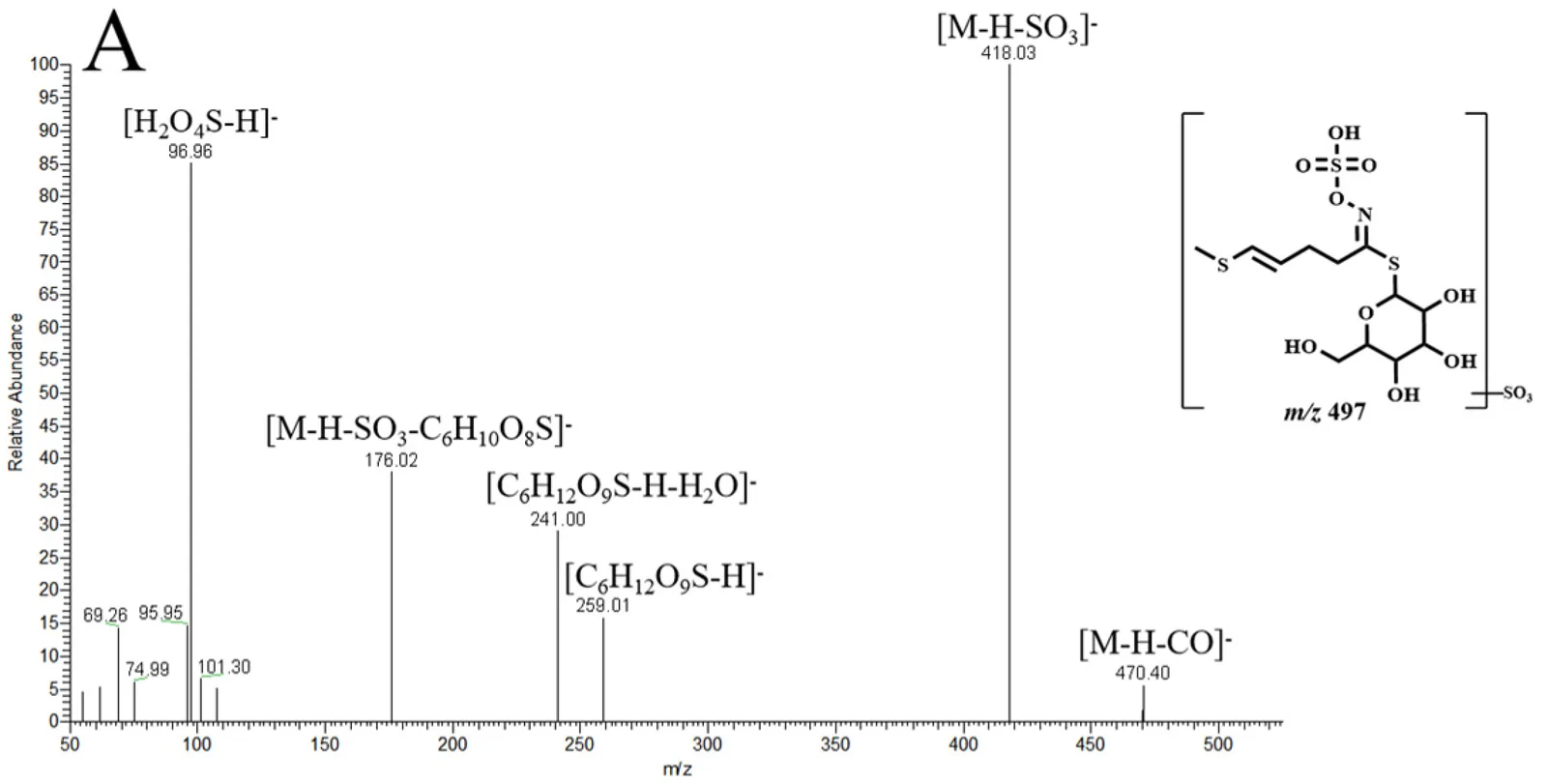

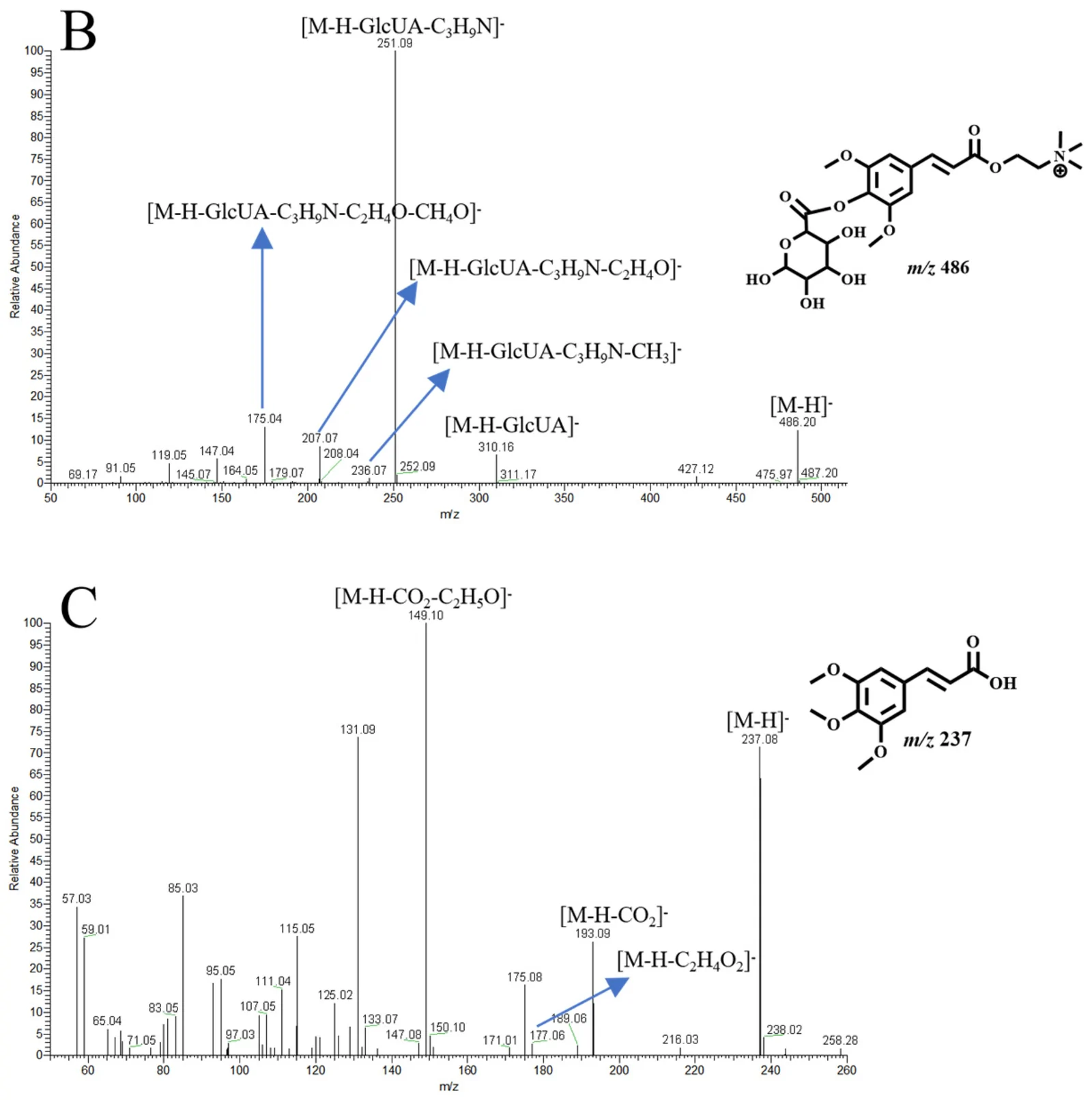
The ESI-MS^2^ spectra and chemical structures of representative metabolites ((**A**): glucoraphasatin sulfation, (**B**): sinapine glucuronidation, (**C**): sinapic acid methylation).

M25 eluted at 3.65 min and generated an [M-H]- ion at *m*/*z* 497.98738. It was observed that the mass increment of 80 Da relative to glucoraphasatin, combined with the TIPIs at *m*/*z* 445 [M-H-SO_3_]^−^, *m*/*z* 259 [M-H-C_6_H_9_O_3_NS_3_]^−^, *m*/*z* 97 [M-H-C_12_H_20_NO_8_S_3_]^−^, and *m*/*z* 80 [M-H-C_12_H_20_NO_9_S_3_]^−^ in its ESI-MS/MS spectrum, suggested sulfation modification. Thus, M25 was tentatively predicted as an isomer of sulfation glucoraphasatin (Figure 7A).

In positive ion mode, M94 produced a [M+H]^+^ ion at *m*/*z* 178.03548 (C_6_H_11_NOS_2_) with a mass error of −0.35 ppm. In its ESI-MS^2^ spectrum, the TIPIs were assigned as *m*/*z* 114 [M+H-CH_4_OS]^−^, *m*/*z* 105 [M+H-C_2_H_3_NS]^−^, *m*/*z* 119 [M+H-CHNS]^−^, *m*/*z* 100 [M+H-C_2_H_8_OS]^−^, and *m*/*z* 119 [M+H-CHNS-C_2_H_4_]^−^. In addition, it was 2 Da more massive than that of sulforaphene, demonstrating that the dihydroxylation reaction might occur. Based on this, M94 was deduced as an isomer of dihydroxylation sulforaphene.

M113 was eluted at 16.00 min and showed a [M+H]^+^ ion at *m*/*z* 162.04056. Its formula was speculated as C_6_H_11_NS_2_ with a mass error of −0.23 ppm. It was 2 Da more massive than that of raphasatin, suggesting that the dihydroxylation reaction occurred. In its ESI-MS^2^ spectrum, the DPIs were assigned as *m*/*z* 114 [M+H-CH_4_S]^+^, *m*/*z* 87 [M+H-C_3_H_7_S]^+^, and *m*/*z* 73 [M+H-C_4_H_9_S]^+^. Thus, M113 was tentatively speculated as an isomer of dihydroxylation raphasatin.

A total of four isomeric metabolites, M40, M50, M54 and M78, displayed [M+H]^+^ ions at *m*/*z* 190.03548. They were 14 Da more massive than that of sulforaphene, and the TIPIs at *m*/*z* 131 [M+H-CHNS]^−^, *m*/*z* 126 [M+H-CH_4_OS]^−^, *m*/*z* 114 [M+H-C_2_H_6_OS]^−^, and *m*/*z* 65 [M+H-C6H7NS_2_]^−^ were observed. Therefore, they were predicted as isomers of methylation sulforaphene. M37 produced the molecular ion at *m*/*z* 192.05113 (C_7_H_13_NOS_2_, mass error of −0.48 ppm) and TIPIs at *m*/*z* 128 [M+H-CH_4_OS]^−^ in positive ion mode, which was 2 Da more massive than that of the above metabolites. Hence, M37 was speculated as an isomer of methylation and dihydroxylation sulforaphene.

M4 was 162 Da more than that of sulforaphene, which gave rise to the [M+H]^+^ ion at *m*/*z* 338.07265 with a mass error of −0.61 ppm and a retention time of 1.26 min. It generated the TIPIs at *m*/*z* 176 [M+H-Glc]^+^ and *m*/*z* 176 [M+H-C_7_H_14_O_6_S]^+^ in its ESI-MS^2^ spectrum. Based on this, M4 was tentatively presumed as an isomer of glucosylation sulforaphene.

M31 generated an [M+H]^+^ ion at *m*/*z* 486.19698 (C_22_H_32_NO_11_, mass error of −0.30 ppm) with a retention time of 5.34 min. It was 176 Da more massive than that of sinapine, implying that it might be the glucuronidation product of sinapine. Fragment ions at *m*/*z* 310 [M+H-GlcUA]^+^, *m*/*z* 251 [M+H-GlcUA-C_3_H_9_N]^+^, *m*/*z* 207 [M+H-GlcUA-C_3_H_9_N-C_2_H_4_O]^+^, and *m*/*z* 175 [M+H-GlcUA-C_3_H_9_N-C_2_H_4_O-CH_4_O]^+^ were exhibited in its ESI-MS^2^ spectra. Hence, M31 was speculated as an isomer of glucuronidation sinapine (Figure 7B).

M41 yielded its significant [M+H]^+^ ion at *m*/*z* 296.14924. The mass decrease of 14 Da compared to sinapine suggested the occurrence of a demethylation reaction. In its ESI-MS/MS spectrum, the TIPIs were observed at *m*/*z* 237 [M+H-C_3_H_9_N]^+^, *m*/*z* 222 [M+H-C_3_H_9_N-CH_3_]^+^, 193 [M+H-C_3_H_9_N-C_2_H_4_O]^+^, 161 [M+H-C_3_H_9_N-C_2_H_4_O-CH_4_O]^+^, and 133 [M+H-C_3_H_9_N-C_2_H_4_O-CH_4_O-CO]^+^. Based on these findings, M41 was tentatively predicted as an isomer of demethylation sinapine. Additionally, M68 produced the molecular ion at *m*/*z* 280.15433 (C_15_H_22_NO_4_, mass error of −0.91 ppm) in positive ion mode, which was 16 Da more massive than that of M41. TIPIs at *m*/*z* 221 [M+H-C_3_H_9_N]^+^, *m*/*z* 206 [M+H-C_3_H_9_N-CH_3_]^+^, *m*/*z* 177 [M+H-C_3_H_9_N-C_2_H_4_O]^+^, *m*/*z* 145 [M+H-C_3_H_9_N-C_2_H_4_O-CH_4_O]^+^, and *m*/*z* 117 [M+H-C_3_H_9_N-C_2_H_4_O-CH_4_O-CO]^+^ were all observed, correspondingly. Hence, M68 was speculated as an isomer of demethylation and deoxidation of sinapine.

M112 generated the molecular [M+H]^+^ ion at *m*/*z* 324.18054 (C_17_H_26_NO_5_, mass error of −0.95 ppm) with a retention time of 15.48 min. It was 14 Da more than that of sinapine, manifesting that a methylation reaction in vivo might have occurred. In its ESI-MS^2^ spectrum, the TIPIs at *m*/*z* 265 [M+H-C_3_H_9_N]^+^, *m*/*z* 250 [M+H-C_3_H_9_N-CH_3_]^+^, *m*/*z* 221 [M+H-C_3_H_9_N-C_2_H_4_O]^+^, *m*/*z* 189 [M+H-C_3_H_9_N-C_2_H_4_O-CH_4_O]^+^, and *m*/*z* 161 [M+H-C_3_H_9_N-C_2_H_4_O-CH_4_O-CO]^+^ were observed. Therefore, M112 was tentatively presumed as an isomer of methylation sinapine. Moreover, M60 was 2 Da more massive than that of M112 and possessed the same fragmentation pattern, which was preliminarily deduced as an isomer of methylation and dihydroxylation sinapine. Similarly, M118 was predicted as an isomer of methylation, dihydroxylation, and deoxygenation sinapine.

A total of 3 isomeric metabolites, M93, M96, and M121, displayed [M-H]^−^ ions at *m*/*z* 237.07684 (C_12_H_14_O_5_, mass error < ±5 ppm) in negative ion mode. They were 14 Da more massive than that of sinapic acid, and the TIPIs at *m*/*z* 193 [M-H-CO_2_]^−^ and *m*/*z* 177 [M-H-C_2_H_4_O_2_]^−^ were observed. Therefore, they were predicted as isomers of methylation sinapic acid (Figure 7C).

M35, M39, M46, and M59 afforded the same theoretical [M-H]^−^ ions at *m*/*z* 303.01801 (C_11_H_12_O_8_S, mass error < ±5 ppm) in negative ion mode. These 4 metabolites were 80 Da more massive than that of sinapic acid, implying that they might be sulfation of sinapic acid. In their ESI-MS^2^ spectra, the TIPIs at *m*/*z* 223 [M-H-SO_3_]^−^, *m*/*z* 208 [M-H-SO_3_-CH_3_]^−^, *m*/*z* 193 [M-H-SO_3_-2CH_3_]^−^, *m*/*z* 163 [M-H-C_2_H_4_O_2_]^−^, *m*/*z* 179 [M-H-CO_2_]^−^, *m*/*z* 149 [M-H-CO_2_-C_2_H_5_]^−^, *m*/*z* 135 [M-H-CO_2_-C_2_H_5_-CH_2_]^−^, and *m*/*z* 121 [M-H-CO_2_-C_2_H_5_-CH_2_O]^−^ were respectively observed in negative ion mode. Thus, they were speculated as isomers of sulfation sinapic acid.

Four isomeric metabolites, M30, M32, M47, and M62, generated [M-H]^−^ ions at *m*/*z* 399.09328, demonstrating a mass shift of+176 Da compared to sinapic acid. Moreover, the TIPIs at *m*/*z* 223 [M-H-GlcUA]^−^, *m*/*z* 208 [M-H-GlcUA-CH_3_]−, *m*/*z* 193 [M-H-GlcUA-2CH_3_]^−^, *m*/*z* 163 [M-H-GlcUA-C_2_H_4_O_2_]^−^, *m*/*z* 179 [M-H-GlcUA-CO_2_]^−^, and *m*/*z* 149 [M-H-GlcUA-CO_2_-C_2_H_5_]^−^ were consistent with glucuronidation derivatives of sinapic acid. Hence, M30, M32, M47, and M62 were predicted as isomers of glucuronidation sinapic acid. According to similar rules, M53, M69, and M82 were speculated as isomers of glucuronidation and de dihydroxylation sinapic acid.

### 2.4. Promoting Gastrointestinal Motility of Raw RS and Stir-Fried RS Decoctions

The promoting gastrointestinal motility of raw RS and stir-fried RS decoctions was evaluated using a visualized zebrafish model. Initially, the physiological tolerability of the interventions was evaluated over a 24 h exposure period to establish appropriate modeling parameters. The concentrations of the drugs were as follows: loperamide hydrochloride (40 μg/mL), domperidone (30 μg/mL), and raw RS and stir-fried RS decoctions (2500 μg/mL, 5000 μg/mL, and 10,000 μg/mL).

**Figure 8:**
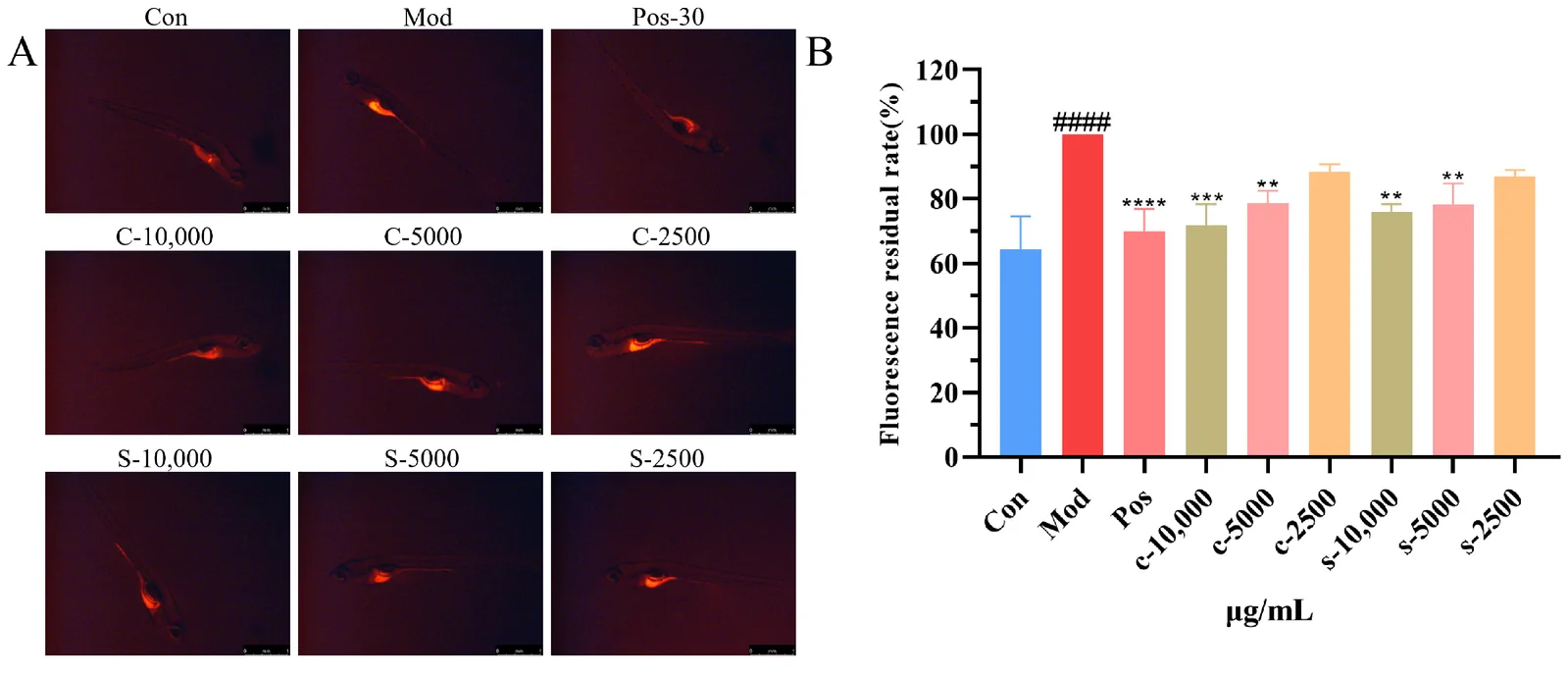
Gastrointestinal motility effect of zebrafish treated with raw RS and stir-fried RS decoctions (*n* = 10). (**A**): Representative gastrointestinal fluorescence phenotypes in zebrafish. (**B**): The promotion of gastrointestinal motility was quantitatively analyzed. Data were expressed as mean ± SD. Compared with Control, ^####^ *p* < 0.0001; compared with model, ** *p* < 0.01, *** *p* < 0.001, **** *p* < 0.0001. (Note: stir-fried RS decoction, S: raw RS decoction).

The promoting gastrointestinal motility of raw RS and stir-fried RS decoctions was visually quantified by monitoring residual fluorescence intensity within the gastrointestinal tract. The experiment conducted a statistical analysis based on the 100% fluorescence residual in the gastrointestinal tract of the model group. The average fluorescence residual rates of the control group and the positive group were 64.4% and 70.2%, respectively. The loperamide-induced model group exhibited intense fluorescence aggregation, indicating that it can delay gastrointestinal emptying. In the positive group, domperidone effectively reduced the fluorescence aggregation, indicating that gastrointestinal emptying was enhanced. At a dose of 10,000 ug/mL of raw RS and stir-fried RS decoctions, the gastrointestinal residual fluorescence levels were 76.3% and 71.8%, and the *p*-values were <0.01 and <0.001, respectively. Moreover, at a dose of 5000 ug/mL, raw RS and stir-fried RS decoctions also exhibited low residual fluorescence levels. The results showed that raw RS and stir-fried RS decoctions possessed the effect of promoting gastrointestinal motility compared with the model group (Figure 8).

### 2.5. Network Pharmacology Analysis [16]

**Figure 9:**
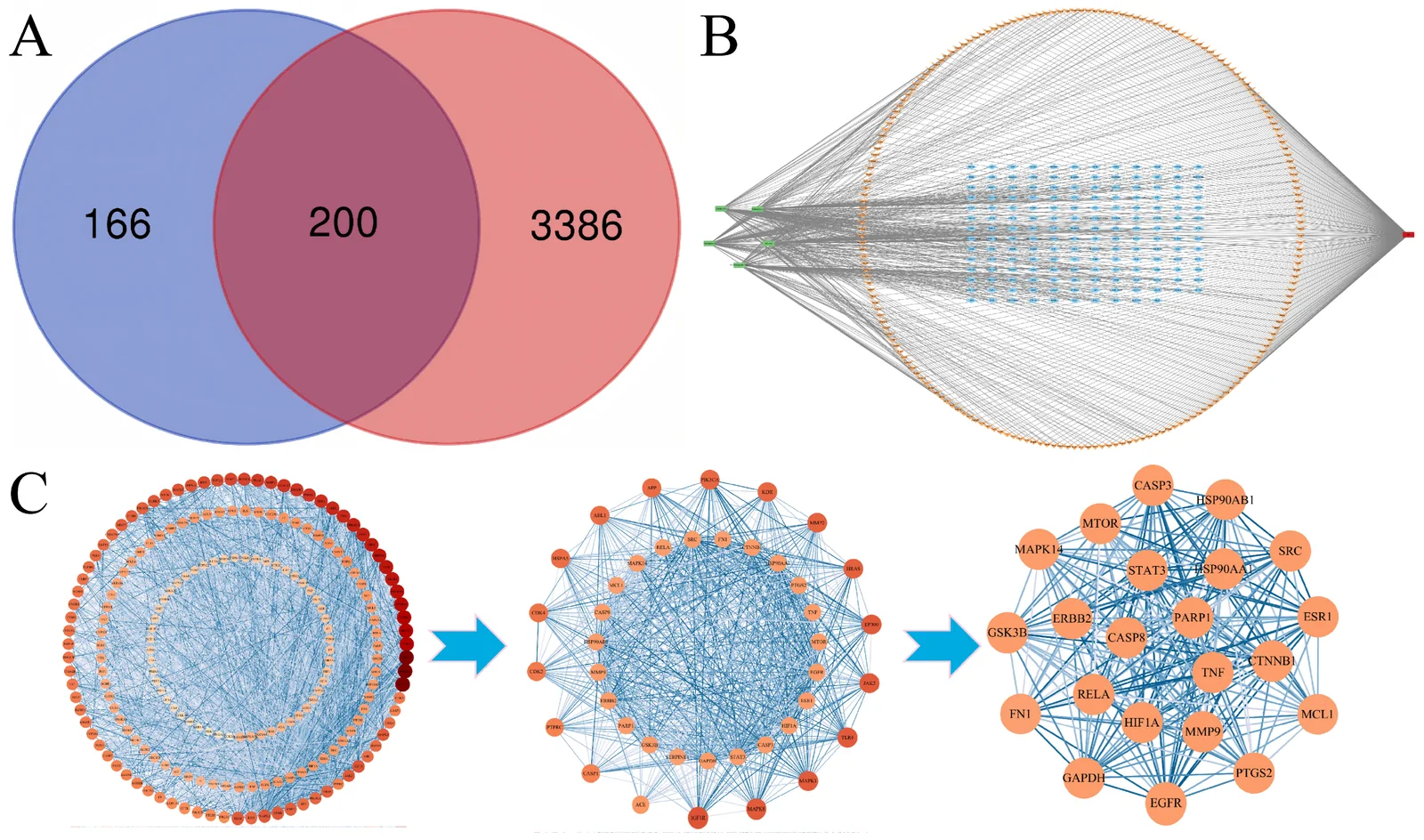
Screening of targets. (**A**): A total of 200 overlapping gene symbols between 5 absorbed compounds and FD, (**B**): disease–target–compounds network, (**C**): interaction network between targets.

In this study, targets of absorbed compounds (glucoraphenin, sulforaphene, sinapine, sinapic acid, and 3,6′-disinapoylsucrose) were selected using the Swiss Target Prediction database. A total of 366 targets were acquired after removing the repeated values. Moreover, 3586 targets of functional dyspepsia (FD) were acquired using GeneCards. Then, 200 common targets were screened by Venn (https://www.bioinformatics.com.cn/static/others/jvenn/example.html, accessed on 23 January 2026) (Figure 9A). The disease–targets–compounds were constructed using Cytoscape 3.10.3 software. There were 372 nodes and 700 edges, among which the red node represented FD and the green nodes represented five main compounds. In addition, orange nodes represented the overlapping targets between FD and the compounds, while the blue nodes represented other targets (Figure 9B).

In total, 199 nodes and 3245 edges in the Protein–Protein Interaction (PPI) network were shown, and the nodes represented the targets and the color represented the degree value of the target. Then, using the median values of degree, closeness, and betweenness as the cut-off values, nodes that simultaneously met these three cut-off values were selected. Then, through CytoNCA in Cytoscape 3.10.3, the core targets of the PPI network were gradually screened out. Finally, 22 core targets, FN1, STAT3, CTNNB1, GAPDH, MCL1, MAPK14, HSP90AA1, ESR1, RELA, PTGS2, SRC, GSK3B, TNF, PARP1, MTOR, EGFR, HIF1A, MMP9, ERBB2, HSP90AB1, CASP8, and CASP3, were screened, manifesting that they may be associated with the treatment of FD (Figure 9C).

**Figure 10:**
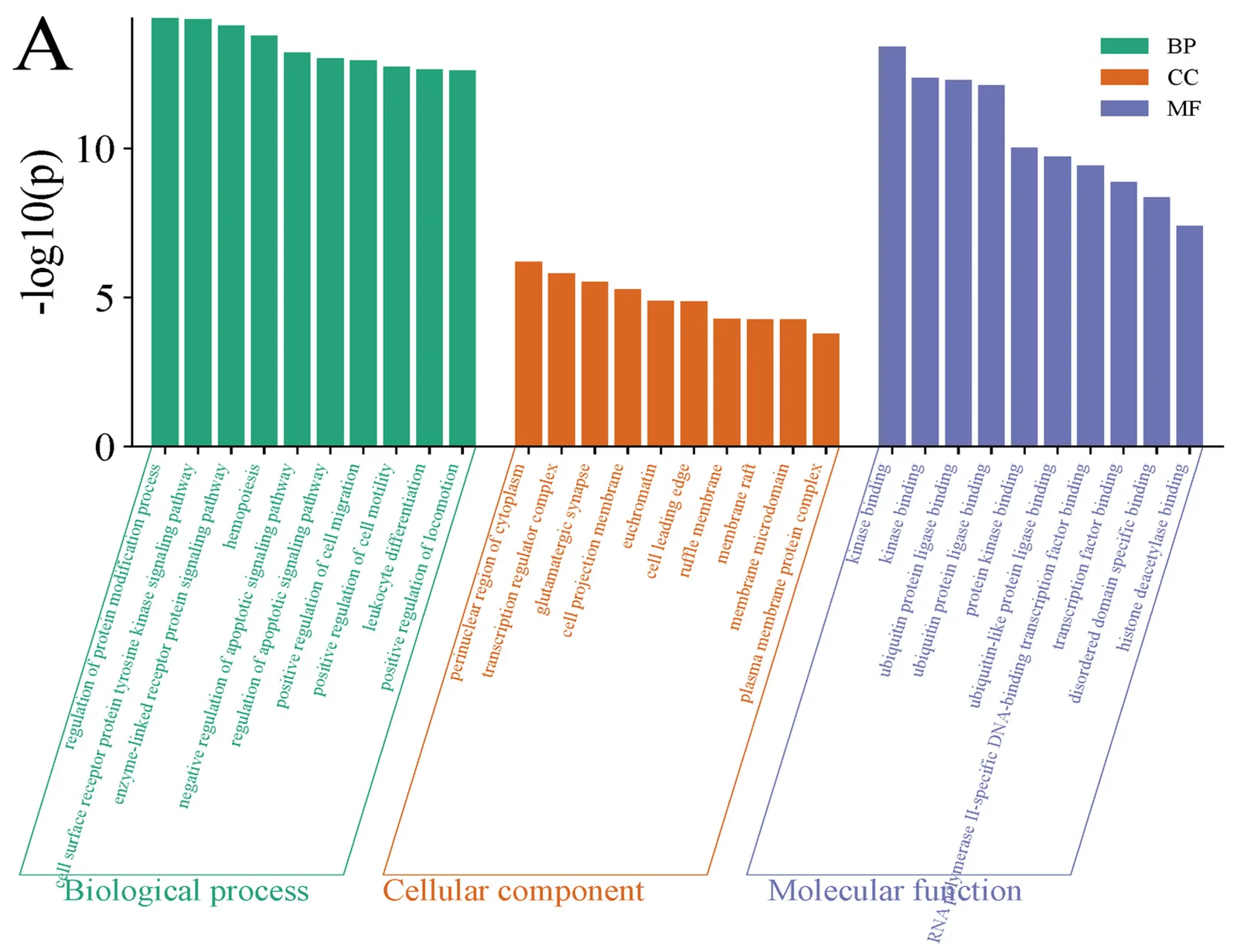

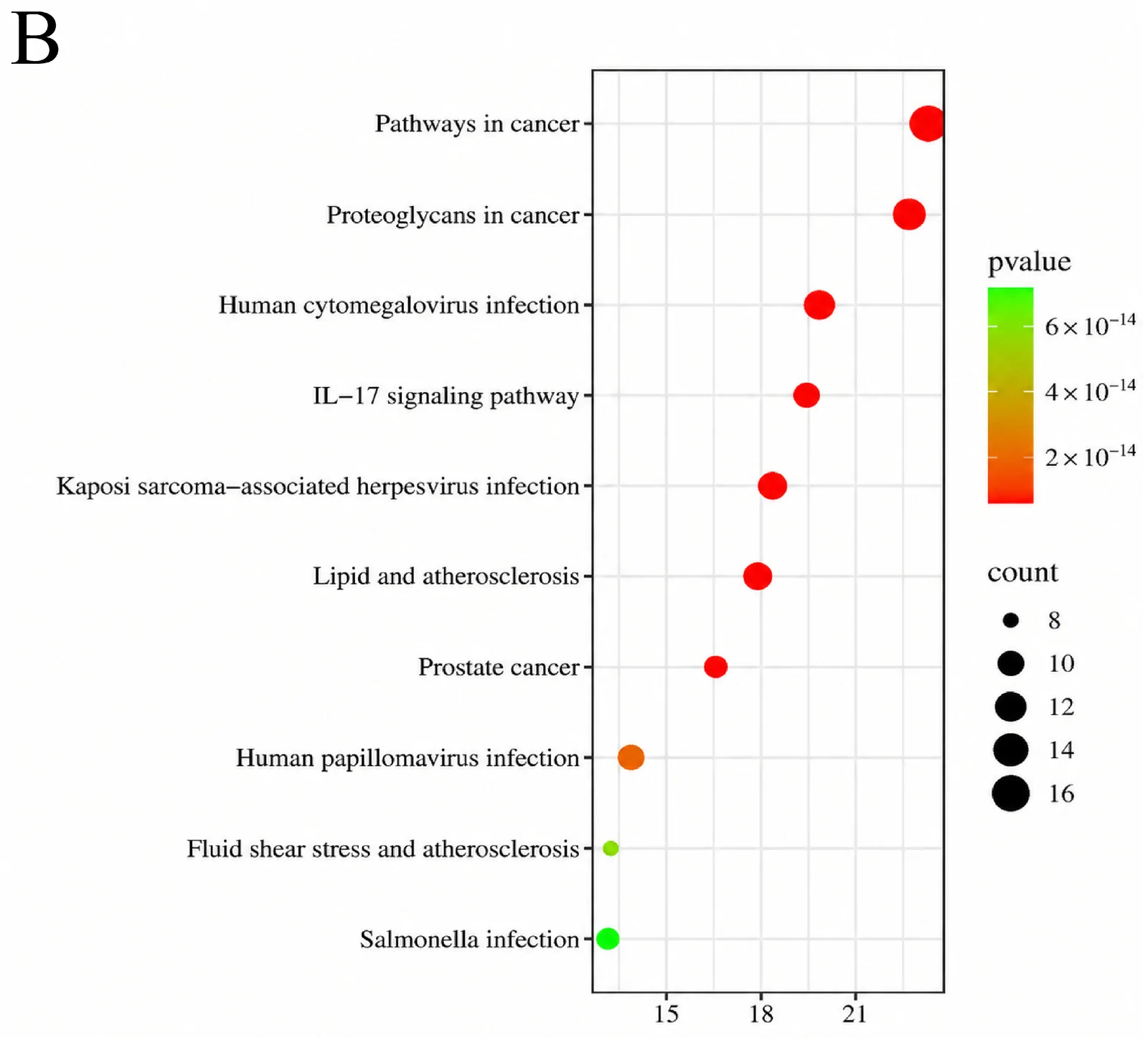
GO (**A**) and KEGG (**B**) analysis.

Then, the above 22 targets were imported into the Metascape database for GO and KEGG enrichment analysis, and *p* < 0.01 pathways were selected. The top 10 biological processes, cellular components, and molecular functions of GO were selected, respectively. The KEGG pathway analysis suggested that pathways in cancer, proteoglycans in cancer, human cytomegalovirus infection, IL-17 signaling pathway, and Kaposi sarcoma-associated herpesvirus infection may be relevant to FD (Figure 10).

### 2.6. Verification of Molecular Docking

**Figure 11:**
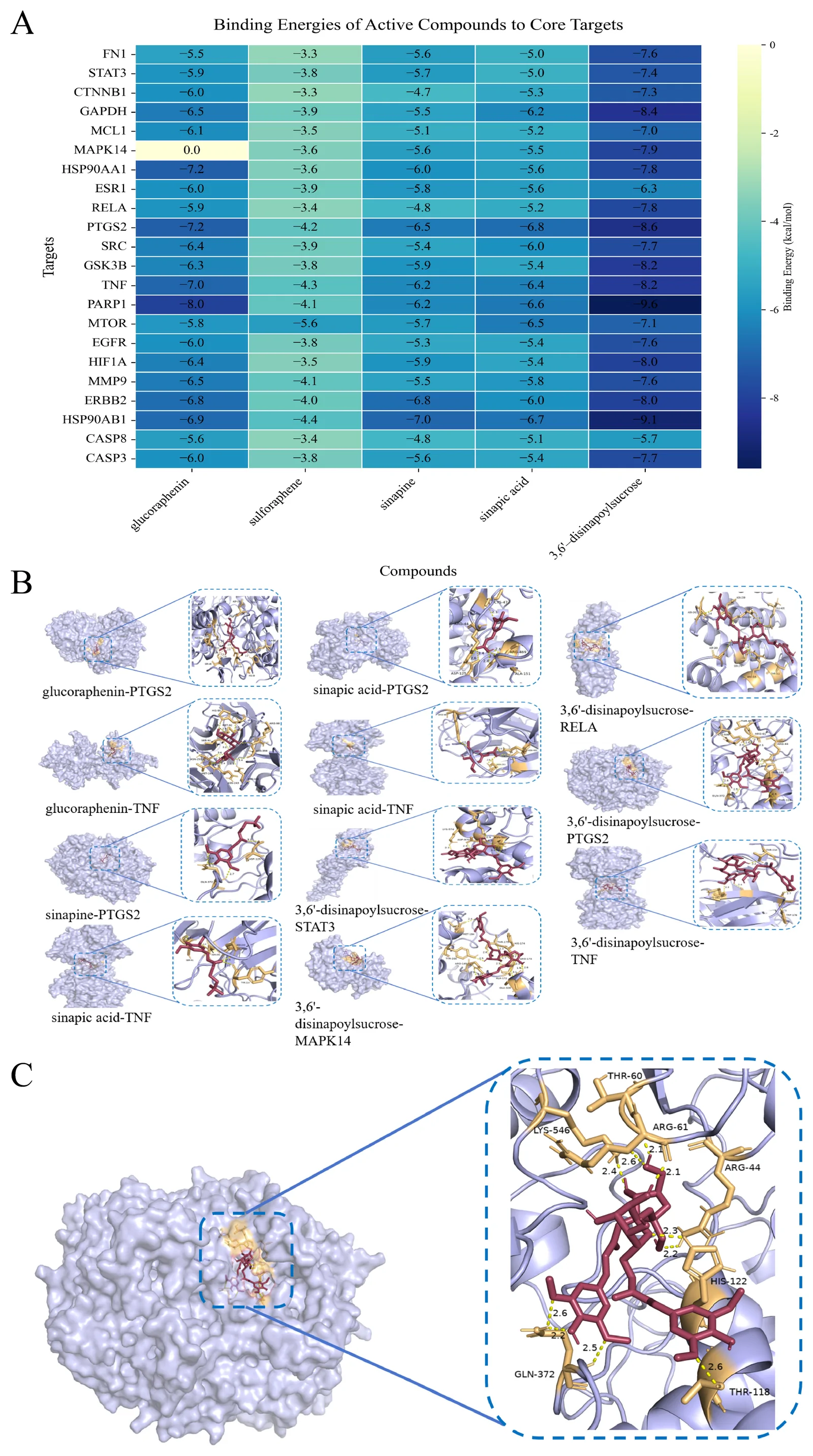
Molecular docking. (**A**): Binding energies of active compounds to core targets, (**B**): Representative compounds-targets, (**C**): Hydrogen-bonding interactions between 3,6′-disinapoylsucrose and PTGS2.

Molecular docking was performed to assess the binding affinities between the five absorbed compounds (glucoraphenin, sulforaphene, sinapine, sinapic acid, and 3,6′-disinapoylsucrose) and targets. Typically, a binding energy of less than −5.0 kcal/mol implies good binding capacity. The comprehensive docking scores are summarized in Figure 11A. The binding energies of glucoraphenin with PTGS2 and TNF were −7.2 kcal/mol and −7.0 kcal/mol, respectively. The binding energy of sulforaphene with mTOR was −5.6 kcal/mol. The binding energy of sinapine with PTGS2 was −6.5 kcal/mol. The binding energies of sinapic acid with PTGS2 and TNF were −6.8 kcal/mol and −6.6 kcal/mol, respectively. Finally, the binding energies of 3,6′-disinapoylsucrose with STAT3, MAPK14, RELA, PTGS2, and TNF were −7.4 kcal/mol, −7.9 kcal/mol, −7.8 kcal/mol, −8.6 kcal/mol, and −8.2 kcal/mol, respectively (Figure 11B). The 3D and 2D of 3,6′-disinapoylsucrose and PTGS2 showed that 3,6′-disinapoylsucrose may successfully occupy the deep active pocket of PTGS2 and it formed stable hydrogen bonds with specific amino acid residues within the binding pocket, including Arg61 (2.1 Å), His122 (2.2 Å and 2.3 Å), Gln372 (2.2 Å, 2.5 Å, and 2.6 Å), Lys546 (2.4 Å and 2.6 Å), and Thr118 (2.6 Å) (Figure 11C).

## 3. Discussion

It was a challenge to elucidate the compounds of raw RS and stir-fried RS decoctions and their metabolites in vivo [17]. To address this issue, based on the fact that compounds with the same or similar core scaffolds during mass spectrometry analysis exhibited similar fragmentation patterns, the TDCSE strategy was constructed [18]. The compounds could yield consistent TIPIs during tandem mass spectrometry (MS/MS). By pairing these TIPIs as diagnostic anchors with predictable precursor mass shifts (Δ *m*/*z*), the TDCSE approach effectively transitions metabolite identification from passive database dependency to active skeleton tracking [19,20]. This database-independent method can reduce the interference from complex endogenous matrices. Ultimately, it facilitated the prediction of novel metabolites, allowing the trajectory of compounds from raw RS and stir-fried RS decoctions to in vivo exposure to be systematically drawn [21,22].

The experiment found that the number of compounds in raw RS decoction was much more than in stir-fried RS decoction, which may be attributable to enzymatic hydrolysis during the decoction of the raw RS to generate their derivatives [23]. Glucosinolates were detected in both raw and stir-fried RS decoctions, whereas isothiocyanates (aglycones of glucosinolates) were detected only in the raw RS decoction. During the decoction of raw RS, the temperature gradually increased. When it reached the optimal temperature for endogenous enzymatic hydrolysis, glucosinolates may be hydrolyzed by enzymes to form isothiocyanates. As the temperature continued to rise, the endogenous enzymes (such as myrosinase) may progressively be inactivated, thereby inhibiting further hydrolysis of glucosinolates [24,25]. In contrast, the high temperatures during stir-frying may inactivate the endogenous enzymes in RS, preventing enzymatic hydrolysis during subsequent decoction preparation. It may be an important factor contributing to the distinct compounds of raw and stir-fried RS decoctions. Moreover, since there were other endogenous enzymes (such as cinnamoyl and octenyl ester enzymes) in RS [26], the phenylpropanoids may also undergo enzymatic reactions in raw RS decoction to generate their derivatives. This factor may also explain why the number of phenylpropanoid derivatives in raw RS decoction is higher than in stir-fried RS decoction. Moreover, the relative peak areas of compounds in raw and stir-fried RS decoctions are shown in Table S1.

Evaluation in a zebrafish model demonstrated that both raw RS and stir-fried RS decoctions possessed the effect of promoting gastrointestinal motility compared with the model group. These findings suggested that the effect of promoting gastrointestinal motility observed in this model was not exclusive to either decoction of RS. Although traditional Chinese medicine stir-frying is generally intended to modify pharmacological effects, reduce toxicity, or optimize therapeutic properties, our findings showed that the effect of promoting gastrointestinal motility was still preserved after stir-frying. Compounds identified through our TDCSE strategy, including glucosinolates (e.g., glucoraphenin) and phenylpropanoid derivatives (e.g., sinapine), may either retain their structural integrity in the stir-fried RS decoction or undergo beneficial enzymatic transformations in the raw RS decoction, which maintained their effect of promoting gastrointestinal motility [27,28,29].

In addition, we speculated on the in vivo metabolic pathways of raw and stir-fried RS decoctions following oral administration to rats. The results showed that glucosinolates and sulfur-containing derivatives and phenylpropanoid derivatives primarily underwent glucuronidation, methylation, sulfation, dihydroxylation, and their composite reactions in vivo. In total, 71 and 59 metabolites were respectively detected in urine and feces, suggesting that metabolites of raw and stir-fried RS decoctions may be eliminated through both urinary and fecal routes. Urinary detection may reflect renal excretion, whereas fecal detection may involve the elimination of metabolites unabsorbed, formed through intestinal biotransformation and excreted into the intestine *via* biliary or other pathways. While sulforaphene was not detected in stir-fried RS decoction, sulforaphene was clearly detected in the plasma of rats after oral administration of stir-fried RS decoction. This might be interpreted as indicating that glucoraphenin can reach the colonic tissue after oral administration to rats and is then slowly decomposed by gut microbiota to generate sulforaphene [30]. Furthermore, other metabolites were detected in plasma that indicated their presence in the systemic circulation following oral administration. These metabolites may serve as candidates for further investigation into the effect of promoting gastrointestinal motility.

In order to further predict the underlying targets and pathways associated with the effect of promoting gastrointestinal motility, network pharmacology and molecular docking were combined. A total of five absorbed compounds—glucoraphenin, sulforaphene, sinapine, sinapic acid, and 3,6′-disinapoylsucrose— were selected as effector molecules for promoting gastrointestinal motility. The results indicated that gastrointestinal dysrhythmia might be driven by inflammatory injury to the interstitial cells of Cajal (ICC) following low-grade mucosal stress [31,32]. Pathway enrichment analysis indicated that the IL-17 signaling pathway might be a regulatory factor in this inflammatory microenvironment [33]. Molecular docking revealed that 3,6′-disinapoylsucrose (−8.6 kcal/mol) and glucoraphenin (−7.2 kcal/mol) exhibited strong binding affinities for the pro-inflammatory enzyme PTGS2, implying that they may have a targeted inhibitory effect and protect ICCs. Owing to its choline ester-like structure, sinapine (−6.5 kcal/mol) may inhibit PTGS2 by interacting with the glutamatergic synaptic nodes and promote intestinal neural conduction [34]. Moreover, smooth muscle propulsion necessitates sustained energy homeostasis, and stress-induced overactivation of PARP1 may deplete cellular ATP pools [35,36].

Sulforaphene exhibited relatively low docking scores, ranging from −3.3 to −5.6 kcal/mol [37]. This apparent discrepancy reflected the inherent limitations of conventional docking algorithms. Because these models exclusively quantified non-covalent interactions, they may underestimate the binding potential of electrophilic small molecules [38]. The interaction between sulforaphene and target proteins (such as mucosal TRP channels) may proceed *via* a distinct two-step process. Initial non-covalent anchoring might align the molecule and facilitate subsequent covalent modification of specific receptor cysteine residues by the reactive isothiocyanate group [39,40]. Consequently, the covalent modulation exerted by these electrophilic compounds may complement the robust non-covalent regulation driven by other bioavailable prototypes. This integration of covalent and non-covalent binding modalities may provide insights into the potential mechanism contributing to the promoting gastrointestinal motility effects of stir-fried RS [41]. It must be pointed out that solid analytical characterization, coupled with network pharmacology and molecular docking, just provided predictive insights into potential therapeutic targets and molecular interactions, rather than definitive evidence elucidating the underlying mechanisms. Therefore, the findings should be regarded as mechanistic hypotheses requiring further experimental validation.

## 4. Materials and Methods

### 4.1. Chemicals and Reagents

Raw RS (Anhui Bozhou Huaishentang Pharmaceutical Co., Ltd., Bozhou, China) was botanically authenticated by Professor Ying Zhang at Jinan University. To avoid variations in compounds caused by batch differences, a portion of the stir-fried RS was prepared in-house using the same batch of raw RS. Sulforaphene (Wuhan Qiongge Biotechnology Co., Ltd., Wuhan, China), glucoraphenin (Chengdu Biopurify Phytochemicals Co., Ltd., Chengdu, China), sibiricose A1, sibiricose A5, sinapine thiocyanate and 3,6′-disinapoylsucrose (Wuhan Zhanzhong Technology Co., Ltd., Wuhan, China), sinapic acid (Yunnan Xili Biotechnology Co., Ltd., Kunming, China) were acquired andtheir purities were acceptable (≥98%) according to HPLC-UV analysis. UHPLC-grade acetonitrile, methanol, and formic acid (FA) were purchased from Thermo Fisher Scientific (Fair Lawn, NJ, USA). Domperidone and loperamide hydrochloride were obtained from Aladdin (Shanghai, China, D129436, L129465). Nile red was purchased from Beijing Xinseng Baite Technology Co., Ltd. (Beijing, China).

### 4.2. Preparation of Decoction of Raw RS and Stir-Fried RS

In accordance with the stir-frying specifications for RS outlined in the 2025 edition of ChP, 100 g of raw RS was stir-fried at a machine temperature of 220–240 °C for 3 min, and raw and stir-fried RS decoctions were sequentially prepared. Briefly, 100 g of either raw or stir-fried RS was mechanically crushed and macerated in 10 volumes of deionized water for 30 min. The raw and stir-fried RS were brought to a vigorous boil, followed by a gentle simmer for 30 min, after which the filtrate was separated and collected. The filter residue was subjected to a secondary exhaustive extraction using 8 volumes of deionized water under identical thermal conditions for 20 min. The pooled decoctions were subsequently concentrated to 1 g/mL and diluted 10-fold for high-resolution mass spectrometric analysis.

### 4.3. Experimental Animals and Biological Sampling

Eighteen male Sprague-Dawley (SD) rats (Guangdong Vital River Laboratory Animal Technology Co., Ltd., Guangzhou, China), 200 ± 10 g, were housed under rigorously controlled environmental conditions (24 ± 2 °C, 70 ± 5% humidity, 12 h light/dark cycle). Following a one-week acclimation, the rats were randomly allocated into three cohorts (*n* = 6 per group): raw RS decoction group, stir-fried RS decoction group, and control group. To ensure strict physiological relevance, the dosage was anchored to a typical human daily intake of 12 g per 60 kg body weight. Utilizing a standard body surface area conversion coefficient of 6.3, the equivalent oral dose for the rats was established at 1.26 g/kg/d. The raw and stir-fried RS decoctions were administered for 3 consecutive days, whereas the control group received an equivalent volume of normal saline. All procedures were approved by the Animal Care and Use Committee of Jinan University (Approval No. 20231122-0001) and adhered strictly to the *Guide for the Care and Use of Laboratory Animals*.

Following a 24 h fasting period (with ad libitum access to water), blood samples (0.5 mL) were longitudinally collected from the suborbital venous plexus into heparin-coated tubes at different time intervals (0.5, 1, 1.5, 2, 4, 8, 12, and 24 h after oral administration). Plasma was isolated *via* centrifugation (3500 rpm, 10 min) and pooled for systemic exposure assessment. Concurrently, 24 h urine and fecal specimens were collected. For sample preparation, 1 mL of plasma or urine was vigorously vortexed with 3 mL of methanol/acetonitrile (1:1, *v*/*v*) for 1 min to precipitate proteins and centrifuged. Lyophilized fecal samples (2 g) were subjected to ultrasonic-assisted extraction in 10 mL of methanol/acetonitrile (1:1, *v*/*v*) for 1 h prior to centrifugation. All supernatants were evaporated to dryness under a gentle nitrogen stream, reconstituted in 300 μL of methanol/acetonitrile (1:1, *v*/*v*), and centrifuged at 14,000 rpm prior to UHPLC-MS/MS injection.

### 4.4. UHPLC-Orbitrap-MS/MS Data Acquisition and Processing

High-resolution chromatographic separation was executed on a DIONEX Ultimate 3000 UHPLC system coupled with a Waters ACQUITY BEH C18 column (2.1 × 100 mm, 1.7 μm). The binary mobile phase consisted of 0.1% FA in water (Solvent A) and acetonitrile (Solvent B). The optimized gradient elution (flow rate: 0.30 mL/min, injection volume: 6 μL) was programmed as follows: 0–10 min, 95% A; 10–15 min, 95%→70% A; 15–32 min, 70%→50% A; 32–35 min, 50%→95% A.

Mass spectrometric analysis was performed using a UHPLC system coupled to a Q Exactive Orbitrap mass spectrometer (Thermo Fisher Scientific) equipped with an electrospray ionization source. Mass spectrometric data were acquired in both positive and negative ion modes. The spray voltage was set to +3.5 kV in positive ion mode and −3.0 kV in negative ion mode. The sheath gas and auxiliary gas flow rates were set to 40 and 20 arbitrary units, respectively. The capillary temperature was maintained at 320 °C, and the probe heater temperature was set to 350 °C. Full MS scans were acquired over an *m*/*z* range of 80–1200 at a resolution of 70,000, with an automatic gain control (AGC) target of 3 × 10^6^ and a maximum injection time of 100 ms. Data-dependent MS^2^ (dd-MS^2^) scans were acquired at a resolution of 35,000, with an AGC target of 1 × 10^5^ and a maximum injection time of 50 ms. Precursor ions with an intensity above the threshold of 1 × 10^5^ were selected for dd-MS^2^ fragmentation. Stepped normalized collision energies (NCEs) of 20, 40, and 60 were applied for dd-MS^2^ acquisition, and the dynamic exclusion duration was set to 6.0 s.

Raw MS data were rigorously interrogated using Thermo Xcalibur 2.1 software. Background noise was systematically eliminated by matrix blank subtraction, applying a three-fold intensity threshold and a retention time tolerance of ±0.2 min. Structural formulas were deduced with a mass tolerance of ±5 ppm, elemental constraints (C 0–60, H 0–90, O 0–60, N 0–30, S 0–20) and corresponding ring double bond (RDB) equivalents of 0–30.

### 4.5. Promoting Gastrointestinal Motility Assessment via Zebrafish Model

Preliminary toxicological evaluations established the maximum tolerance concentration (MTC) to ensure physiological relevance. Morphologically normal larvae at 5 days post-fertilization (dpf) were selected following standard husbandry protocols. Larvae (5–6 dpf, *n* = 10/well) were exposed to different concentrations of raw RS decoction, stir-fried RS decoction, loperamide, and domperidone in 12-well microplates for 24 h, and survival rates were recorded. In total, 3 independent repeated experiments were conducted.

Larvae were allocated into 9 groups: control group, model group (loperamide, 40 μg/mL), positive group (domperidone, 30 μg/mL), and 6 intervention groups receiving specified concentrations (low: 2500 μg/mL; medium: 5000 μg/mL; high: 10,000 μg/mL) of raw or stir-fried RS decoctions. The gastrointestinal tract was visually tracked *via* a 16 h pre-incubation with Nile red (10 μg/mL). Subsequently, loperamide (40 μg/mL) was administered to induce gastrointestinal peristalsis impairment across all test groups (excluding the control group). Following a 24 h co-incubation with the respective treatments in the dark at 28 °C, larvae were anesthetized with 0.02% tricaine and immobilized in 3% methylcellulose. To assess the effect of promoting gastrointestinal motility, a fluorescence microscope was employed to record the gastrointestinal fluorescent signals of larvae from each group. Quantitative evaluation of gastrointestinal fluorescence intensity (S) was facilitated by ImageJ software (version 1.53). In total, 3 independent repeated experiments were conducted. The promoting gastrointestinal motility was determined utilizing the formula below:

fluorescence residual rate (%) = S_0_/S

PS: S represents the average gastrointestinal fluorescence intensity of all surviving larvae in the model group; S_0_ represents the individual original fluorescence signal intensity of each observed larva (including control group, model group, and treatment groups).

### 4.6. Network Pharmacology

Canonical SMILES of 5 absorbed compounds (glucoraphenin, sulforaphene, sinapine, sinapic acid, and 3,6′-disinapoylsucrose) were retrieved from the PubChem database, and their “Homo sapiens” targets were predicted using Swiss Target Prediction (http://www.swisstargetprediction.ch/, 23 January 2026). Disease-associated targets for FD were acquired from the GeneCards (http://www.genecards.org/, 23 January 2026) database. Potential therapeutic targets were predicted by intersecting the compound-related targets with the FD-related targets. A PPI network for these shared targets was constructed *via* the STRING database (https://cn.string-db.org/, 25 January 2026), and core targets were subsequently screened based on topological degree values using Cytoscape 3.10.3. Functional enrichment analysis of Gene Ontology (GO) and Kyoto Encyclopedia of Genes and Genomes (KEGG) pathways was performed using Metascape with a significance threshold of *p* < 0.01. Finally, a disease–targets–compounds network was constructed and visualized using Cytoscape 3.10.3 to explore potential pharmacological relationships.

### 4.7. Molecular Docking

The 3D structures of FN1, STAT3, CTNNB1, GAPDH, MCL1, MAPK14, HSP90AA1, ESR1, RELA, PTGS2, SRC, GSK3B, TNF, PARP1, MTOR, EGFR, HIF1A, MMP9, ERBB2, HSP90AB1, CASP8, and CASP3 were retrieved from the RCSB Protein Data Bank (RCSB PDB) and prepared in PyMOL 3.1.7.1 by removing water molecules, heteroatoms, and co-crystallized ligands. Glucoraphenin, sulforaphene, sinapine, sinapic acid, and 3,6′-disinapoylsucrose structures were obtained from PubChem and energy-minimized in Chem3D. Both the receptors and ligands were processed in AutoDock Tools 1.5.6 to add polar hydrogens and Gasteiger charges, and to define rotatable bonds for the ligands. Then, they were saved as PDBQT files. Semi-flexible docking was performed using AutoDock Vina with an exhaustiveness of 5. The pose with the lowest binding affinity (kcal/mol) was selected for 3D visualization and non-covalent interaction analysis using PyMOL 3.1.7.1.

### 4.8. Statistical Analysis

Statistical analyses were performed using one-way analysis of variance (ANOVA) to evaluate differences among multiple groups. All results were presented as the mean ± standard deviation (SD), with statistical significance defined as *p* < 0.05.

## 5. Conclusions

This study employed the TDCSE strategy to clarify the compounds in raw RS and stir-fried RS decoctions and their metabolites in vivo. A total of 26 compounds were detected in stir-fried RS decoction. In addition to these 26 compounds, another 50 compounds were detected in raw RS decoction. Moreover, a total of 123 metabolites (including prototypes) were detected after removing duplicates. In total, 18, 71, 59, and 4 metabolites were discovered in plasma, urine, feces, and liver tissue, respectively. The zebrafish model was used to evaluate the promoting gastrointestinal motility of raw RS and stir-fried RS decoctions. The results showed that both raw RS and stir-fried RS decoctions possessed the effect of promoting gastrointestinal motility compared with the model group. The network pharmacology and molecular docking techniques were used to predict potential targets and pathways of stir-fried RS decoctions that promote gastrointestinal motility. Potential targets, PTGS2, TNF, PARP1, MTOR, etc., and the IL-17 pathway were predicted. Overall, this study clarified the differences in compounds and in vivo metabolites of raw and stir-fried RS decoctions. It provided a reference for further investigation into the effect of promoting gastrointestinal motility of RS.

## Data Availability

Most of the data used during the preparation of the manuscript are included in the Results and Discussion sections. However, for any additional details of the procedures and the original raw files, please contact the corresponding authors.

## Supplementary Materials

The following supporting information can be downloaded at: https://www.mdpi.com/article/10.3390/molecules31213942/s1, Table S1: Compounds of raw and stir-fried RS decoctions; Table S2: Metabolites in vivo of raw and stir-fried RS decoctions; Figure S1: The chromatogram of raw and stir-fried RS decoctions (A: negative ion mode of raw RS decoction, B: positive ion mode of raw RS decoction, C: negative ion mode of stir-fried RS decoction, D: positive ion mode of stir-fried RS decoction); Figure S2: The chromatogram of raw and stir-fried RS decoctions in plasma (A: negative ion mode of raw RS decoction, B: positive ion mode of raw RS decoction, C: negative ion mode of stir-fried RS decoction, D: positive ion mode of stir-fried RS decoction); Figure S3: The chromatogram of raw and stir-fried RS decoctions in urine (A: negative ion mode of raw RS decoction, B: positive ion mode of raw RS decoction, C: negative ion mode of stir-fried RS decoction, D: positive ion mode of stir-fried RS decoction); Figure S4: The chromatogram of raw and stir-fried RS decoctions in feces (A: negative ion mode of raw RS decoction, B: positive ion mode of raw RS decoction, C: negative ion mode of stir-fried RS decoction, D: positive ion mode of stir-fried RS decoction). Figure S5. The chromatogram of raw and stir-fried RS decoctions in liver tissue (A: negative ion mode of raw RS decoction, B: positive ion mode of raw RS decoction, C: negative ion mode of stir-fried RS decoction, D: positive ion mode of stir-fried RS decoction).

## References

[1] Li Y., Lei Z., Guo Y., Liu Y., Guo X., Wang X., Che J., Yuan J., Wang C., Li M. (2023). Fermentation of Ganoderma lucidum and Raphani Semen with a probiotic mixture attenuates cyclophosphamide-induced immunosuppression through microbiota-dependent or -independent regulation of intestinal mucosal barrier and immune responses. Phytomedicine.

[2] Gao L., Li H., Li B., Shao H., Yu X., Miao Z., Zhang L., Zhu L., Sheng H. (2022). Traditional uses, phytochemistry, transformation of ingredients and pharmacology of the dried seeds of *Raphanus sativus* L. (Raphani Semen), A comprehensive review. J. Ethnopharmacol..

[3] Yu X., Gao L., Miao Z., Zhang L., Wu R., Sun S., Sun N., Zhu L., Sheng H. (2023). Qualitative and quantitative analysis of the component variations of Raphani Semen during the stir-frying process and elucidation of transformation pathways of multiple components. J. Pharm. Biomed. Anal..

[4] Gao S., Wang J., Cheng L., Fan Y., Qin W., Wang Y., Guo Y., Zhang X., Yang Y. (2022). Evaluation of the Effects of Processing Technique on Chemical Components in Raphani Semen by HPLC and UPLC-Q-TOF-MS. Int. J. Anal. Chem..

[5] Lv W., Ren T., Su Y., Meng X. (2011). Inhibition of glucoraphenin enzymolysis in Raphani Semen by processing. Zhongguo Zhong Yao Za Zhi.

[6] Wang M., Carver J.J., Phelan V.V., Sanchez L.M., Garg N., Peng Y., Nguyen D.D., Watrous J., Kapono C.A., Luzzatto-Knaan T. (2016). Sharing and community curation of mass spectrometry data with Global Natural Products Social Molecular Networking. Nat. Biotechnol..

[7] Nothias L.F., Petras D., Schmid R., Dührkop K., Rainer J., Sarvepalli A., Protsyuk I., Ernst M., Tsugawa H., Fleischauer M. (2020). Feature-based molecular networking in the GNPS analysis environment. Nat. Methods.

[8] Lan X., Song S., Zhang Z., Yuan X., Wang H., Mei X., Cai W., Zhang J. (2025). Mass Spectrometry Fragmentation Recursion Tree coupled with Diagnostic Product Ions Strategy for comprehensive characterization of secondary and in vivo metabolites of Astragali Radix. Talanta.

[9] Rich A. (2018). Improved Imaging of Zebrafish Motility. Neurogastroenterol. Motil..

[10] Cassar S., Huang X., Cole T. (2018). High-throughput Measurement of Gut Transit Time Using Larval Zebrafish. J. Vis. Exp..

[11] Nogales C., Mamdouh Z.M., List M., Kiel C., Casas A.I., Schmidt H. (2022). Network pharmacology: Curing causal mechanisms instead of treating symptoms. Trends Pharmacol. Sci..

[12] Zhang R., Zhu X., Bai H., Ning K. (2019). Network Pharmacology Databases for Traditional Chinese Medicine: Review and Assessment. Front. Pharmacol..

[13] Crampon K., Giorkallos A., Deldossi M., Baud S., Steffenel L.A. (2022). Machine-learning methods for ligand-protein molecular docking. Drug Discov. Today.

[14] Cui Y.F., Zhang W.W., Li Y.N., Xu J., Lan X.M., Song S.Y., Lin Y.Q., Dai L., Zhang J.Y. (2022). The Analytical Strategy of “Ion Induction and Deduction Based on Net-Hubs” for the Comprehensive Characterization of Naringenin Metabolites In Vivo and In Vitro Using a UHPLC-Q-Exactive Orbitrap Mass Spectrometer. Molecules.

[15] Yuan X., Wang H., Song S., Qiu L., Lan X., Dong P., Zhang J. (2023). Stepwise Targeted Matching Strategy for Comprehensive Profiling of Xanthohumol Metabolites In Vivo and In Vitro Using UHPLC-Q-Exactive Orbitrap Mass Spectrometer. Molecules.

[16] Li M., Xu Y., Yang M., Jiang X., Liu Y., Lei L., Lu X., Wan X., Yang Y. (2026). Screening the effective components of Ficus tikoua on the treatment of lipopolysaccharide induced acute lung injury mice by integrated UHPLC-Q-TOF-MS/MS and network pharmacology. J. Pharm. Biomed. Anal..

[17] Ting Y.S., Egertson J.D., Payne S.H., Kim S., MacLean B., Käll L., Aebersold R., Smith R.D., Noble W.S., MacCoss M.J. (2015). Peptide-Centric Proteome Analysis: An Alternative Strategy for the Analysis of Tandem Mass Spectrometry Data. Mol. Cell. Proteom..

[18] Zhang H., Bensaddek D. (2025). Optimized Time-Segmented Acquisition Expands Peptide and Protein Identification in TIMS-TOF Pro Mass Spectrometry. J. Proteome Res..

[19] Lan X., Li Y., Li H., Song S., Yuan X., Zhou H., Chen Q., Zhang J. (2022). Drug metabolite cluster centers-based strategy for comprehensive profiling of Neomangiferin metabolites in vivo and in vitro and network pharmacology study on anti-inflammatory mechanism. Arab. J. Chem..

[20] Song S., Li Y., Liu X., Yu J., Li Z., Liang K., Wang S., Zhang J. (2023). Study on the Biotransformation and Activities of Astragalosides from Astragali Radix In Vitro and In Vivo. J. Agric. Food Chem..

[21] Covington B.C., McLean J.A., Bachmann B.O. (2017). Comparative mass spectrometry-based metabolomics strategies for the investigation of microbial secondary metabolites. Nat. Prod. Rep..

[22] Zhou H., Song S., Lan X., Li Y., Yuan X., Yang J., Li M., Cao T., Zhang J. (2023). Comprehensive Profiling of Mangiferin Metabolites In Vivo and In Vitro Based on the “Drug Metabolite Clusters” Analytical Strategy. ACS Omega.

[23] Wu X., Huang H., Childs H., Wu Y., Yu L., Pehrsson P.R. (2021). Glucosinolates in Brassica Vegetables: Characterization and Factors That Influence Distribution, Content, and Intake. Annu. Rev. Food Sci. Technol..

[24] Rungapamestry V., Duncan A.J., Fuller Z., Ratcliffe B. (2006). Changes in glucosinolate concentrations, myrosinase activity, and production of metabolites of glucosinolates in cabbage (*Brassica oleracea* Var. capitata) cooked for different durations. J. Agric. Food Chem..

[25] Ludikhuyze L., Ooms V., Weemaes C., Hendrickx M. (1999). Kinetic study of the irreversible thermal and pressure inactivation of myrosinase from broccoli (*Brassica oleracea* L. Cv. italica). J. Agric. Food Chem..

[26] Nićiforović N., Abramovič H. (2014). Sinapic Acid and Its Derivatives: Natural Sources and Bioactivity. Compr. Rev. Food Sci. Food Saf..

[27] Martinez J.M., Eling T.E. (2019). Activation of TRPA1 by volatile organic chemicals leading to sensory irritation. Altex.

[28] Evans C., Howells K., Suzuki R., Brown A.J.H., Cox H.M. (2023). Regional characterisation of TRPV1 and TRPA1 signalling in the mouse colon mucosa. Eur. J. Pharmacol..

[29] Yang G., Bai C., Liu T., He J., Gu X. (2021). The effect of *Raphanus sativus* L. seeds on regulation of intestinal motility in rats consuming a high-calorie diet. Biomed. Pharmacother..

[30] Narbad A., Rossiter J.T. (2018). Gut Glucosinolate Metabolism and Isothiocyanate Production. Mol. Nutr. Food Res..

[31] Sanders K.M., Ward S.M., Koh S.D. (2014). Interstitial cells: Regulators of smooth muscle function. Physiol. Rev..

[32] Sanders K.M., Santana L.F., Baker S.A. (2026). Interstitial cells of Cajal—Pacemakers of the gastrointestinal tract. J. Physiol..

[33] Chen L., Ruan G., Cheng Y., Yi A., Chen D., Wei Y. (2022). The role of Th17 cells in inflammatory bowel disease and the research progress. Front. Immunol..

[34] Zhang Z., Guo J., Huang Z., Zheng X., Xiong P. (2026). Exploring the Treatment of Cinnamomum Cassia Leaf Extract in Ulcerative Colitis: Network Pharmacology and In Vitro Investigations. Plants.

[35] Maluchenko N.V., Feofanov A.V., Studitsky V.M. (2021). PARP-1-Associated Pathological Processes: Inhibition by Natural Polyphenols. Int. J. Mol. Sci..

[36] Ba X., Garg N.J. (2011). Signaling mechanism of poly(ADP-ribose) polymerase-1 (PARP-1) in inflammatory diseases. Am. J. Pathol..

[37] Manivannan A., Kim J.H., Kim D.S., Lee E.S., Lee H.E. (2019). Deciphering the Nutraceutical Potential of Raphanus sativus-A Comprehensive Overview. Nutrients.

[38] Russo M., Spagnuolo C., Russo G.L., Skalicka-Woźniak K., Daglia M., Sobarzo-Sánchez E., Nabavi S.F., Nabavi S.M. (2018). Nrf2 targeting by sulforaphane: A potential therapy for cancer treatment. Crit. Rev. Food Sci. Nutr..

[39] Abassi P., Abassi F., Yari F., Hashemi M., Nafisi S. (2013). Study on the interaction of sulforaphane with human and bovine serum albumins. J. Photochem. Photobiol. B.

[40] Nguyen V.P.T., Stewart J.D., Ioannou I., Allais F. (2021). Sinapic Acid and Sinapate Esters in Brassica: Innate Accumulation, Biosynthesis, Accessibility via Chemical Synthesis or Recovery From Biomass, and Biological Activities. Front. Chem..

[41] Mi L., Di Pasqua A.J., Chung F.L. (2011). Proteins as binding targets of isothiocyanates in cancer prevention. Carcinogenesis.

