# Supplementary material for: Taxonomy-Driven Core Scaffold Expansion Strategy for Comprehensive Profiling of Secondary and In Vivo Metabolites of Raphani Semen and Study on Promoting Gastrointestinal Motility
Source: Molecules. 2026 Nov 2;31(21):3942. doi: 10.3390/molecules31213942 (PMC13765078; doi:10.3390/molecules31213942)
Supplement: Supplementary file 1 [file molecules-31-03942-s001.zip › molecules-4609425-supplementary.pdf]

Table S1. Compounds of raw and stir-fried RS decoctions.

NO.	Rt (min)	compound	Formula	Theoretical (m/z)	Experiment (m/z)	RDB	Error (ppm)	Fragment ions	ion mode	Relative peak area ($\times 10^4$)	
										S	C
A1	1.00	glucoraphenin	$C_{12}H_{21}NO_{10}S_3$	434.02548	434.02542	3.5	-0.14	97(100.0),434(47.8),75(33.0),80(13.3),419(3.5),259(3.0),192(1.1)	N	1699.2	59472.0
A2	1.14	4-methylsulfonyl-3-butenyl glucosinolate isomer	$C_{12}H_{21}NO_{11}S_3$	450.02039	450.02020	3.5	2.00	97(100.0),450(39.6),75(32.6),80(22.6),386(12.4),192(6.8),259(2.8),435(1.7)	N	12.8	65.9
A3	1.17	glucoalyssin isomer	$C_{13}H_{25}NO_{10}S_3$	450.05678	450.05667	2.5	-0.25	97(100.0),450(39.6),75(32.6),386(12.4),192(6.8),259(2.8),435(1.7)	N	15.5	151.4
A4	1.66	hydroxycinnamic acid isomer	$C_9H_8O_3$	163.04006	163.03928	6.5	-4.83	119(100.0),163(9.2)85(3.1),59(2.6),93(1.0),71(0.9),89(0.7)	N	14.4	-
A5	1.70	gluconapin isomer	$C_{11}H_{19}NO_9S_2$	372.04284	372.04282	3.5	-0.07	97(100.0),75(73.7),372(64.8),259(2.9)130(2.7),195(2.4)	N	12.3	105.4
A6	1.89	5-(methylsulfinyl)-4-pentenenitrile	C_6H_9NOS	144.04776	144.04767	2.5	-0.63	87(100.0),144(88.2),98(44.9),104(28.8),53(20.2),89(12.8),55(7.9),65(7.9)	P	159275.6	-
A7	2.13	6-[(methylsulfinyl)methyl]-1,3-thiazinan-2-thione isomer	$C_6H_{11}NOS_3$	210.00755	210.00740	1.5	-0.73	146(100.0),87(78.2),85(4.0),59(2.3),210(1.8),53(1.2),147(1.1)	P	241.6	57953.3
A8	2.25	3-methylthiopropyl-Glucosinolate isomer	$C_{11}H_{21}NO_9S_3$	406.03056	406.03085	2.5	0.70	97(100),406(74.0),75(49.4),80(14.7),153(11.1),167(9.0),183(8.8)	N	22.8	64.1
A9	2.39	4-hydroxyglucobrassicin isomer	$C_{16}H_{20}N_2O_{10}S_2$	463.04865	463.04889	8.5	0.50	97(100.0),75(21.5),463(15.8),80(6.1),169(5.2),267(4.5),285(2.7),221(1.8),313(1.4),259(1.3)	N	70.8	2064.4
A10	2.41	6-[(methylsulfinyl)methyl]-1,3-thiazinan-2-thione isomer	$C_6H_{11}NOS_3$	210.00755	210.00742	1.5	-0.63	146(100.0),87(72.2),118(11.3),72(5.1),85(3.2),59(1.8),210(1.5)	P	97.6	41467.9

A11	2.70	4-hydroxyglucobrassicin isomer	$C_{16}H_{20}N_2O_{10}S_2$	463.04865	463.04889	8.5	0.50	97(100.0),75(21.0),463(15.4),267(4.8),169(4.3),285(3.3),259(1.4),221(1.4)	N	55.4	2449.4
A12	4.41	4-methylthiobutyl glucosinolate isomer	$C_{12}H_{23}NO_9S_3$	420.04621	420.04633	2.5	0.27	97(100),96(59.8),420(58.2),75(47.8),80(13.7),195(2.3),85(1.9),259(1.8),71(1.7)	N	62.3	298.3
A13	4.92	4-hydroxyphenyllactic acid isomer	$C_9H_{10}O_4$	181.05063	181.05002	5.5	-3.38	163(100.0),137(88.0),181(74.7),119(57.6),73(35.1),89(10.7)	N	21.5	-
A14	5.06	glucoraphasatin isomer	$C_{12}H_{21}NO_9S_3$	418.03056	418.03076	3.5	0.46	97(100.0),418(63.1),96(49.0),75(46.0),80(16.3),69(6.2),59(3.7),176(3.1),195(2.9),103(2.3)	N	482.9	12993.3
A15	5.17	4-methylthiobutyl glucosinolate isomer	$C_{12}H_{23}NO_9S_3$	420.04621	420.04633	2.5	0.27	97(100.0),420(60.3),75(51.8),80(15.5),259(2.5),178(1.4),195(1.3)	N	623.1	1849.2
A16	5.23	2-propenyl glucosinolate isomer	$C_{10}H_{17}NO_9S_2$	358.02719	358.02377	3.5	-3.51	132(100.0),278(34.8),88(24.2),358(12.8),115(10.7),97(6.3),145(5.7),307(2.9)	N	66.0	-
A17	5.24	glucorapiferin isomer	$C_{11}H_{19}NO_{10}S_2$	388.03776	388.03445	3.5	-4.70	308(100.0),146(78.9),128(53.3),161(46.6),388(35.3),135(32.4),97(23.7)	N	30.2	-
A18	6.41	caffeic acid hexoside isomer	$C_{15}H_{18}O_9$	341.08780	341.08759	7.5	-0.63	179(100.0),281(79.7),135(73.8),161(71.7),251(46.0),221(40.9),177(32.0),341(8.3)	N	202.6	-
A19	6.82	[(2R,3S,4S,5R,6R)-6-[1,7-bis(4-hydroxyphenyl)-5-oxoheptan-3-yl]oxy-3,4,5-trihydroxyoxan-2-yl]methyl (E)-3-(4-hydroxyphenyl)prop-2-enoate isomer	$C_{34}H_{38}O_{11}$	621.23413	621.23016	16.5	-3.40	137(100.0),93(48.1),299(25.6),621(10.0)	N	25.6	-

A20	6.83	2-(4-hydroxy-1H-indol-3-yl)acetonitrile isomer	$C_{10}H_8N_2O$	173.07093	173.07079	7.5	-0.86	133(100.0),146(27.6),173(24.2),118(7.8),164(4.4),145(4.0),105(2.8),130(1.5),91(1.4)	P	257.0	-
A21	7.01	3-methylbutyl glucosinolate isomer	$C_{12}H_{23}NO_9S_2$	388.07414	388.07413	2.5	2.79	97(100.0),388(94.5),75(83.3),80(14.4),59(6.4),259(4.7),101(4.5)	N	13.3	-
A22	7.29	glucobrassicin isomer	$C_{16}H_{20}N_2O_9S_2$	447.05374	447.05380	8.5	0.12	97(100.0),447(47.6),75(40.3),80(13.9),259(2.4)	N	100.2	-
A23	7.47	3-methylbutyl glucosinolate isomer	$C_{12}H_{23}NO_9S_2$	388.07414	388.07413	2.5	2.79	97(100.0),388(99.9),75(68.1),80(12.5),89(5.5)	N	103.3	-
A24	7.47	6-O-(e)-p-coumaroyl glucopyranose isomer	$C_{15}H_{18}O_8$	325.09289	325.09274	7.5	-0.46	145(100.0),187(59.0),119(31.8),325(31.1),163(29.0),265(6.8)	N	47.6	-
A25	7.54	chlorogenic acid hemihydrate isomer	$C_{16}H_{20}O_{10}$	371.09837	371.09827	7.5	-0.27	311(100.0),209(71.1),281(52.1),191(45.1),251(40.6),207(18.1),371(17.6),165(16.0)	N	168.7	-
A26	7.57	caffeic acid hexoside isomer	$C_{15}H_{18}O_9$	341.08780	341.08765	7.5	-0.46	281(100.0),179(92.1),135(78.5),161(78.1),251(64.2),221(52.9),341(19.0)	N	329.5	-
A27	7.99	4-coumaroylcholine isomer	$C_{14}H_{20}NO_3$	250.14377	250.14354	5.5	-0.92	191(100.0),147(50.3),250(11.4),119(7.5),91(1.2),251(0.6),120(0.3)	P	622.2	20496.6
A28	8.40	isofraxidin isomer	$C_{11}H_{10}O_5$	221.04554	221.04504	7.5	-2.29	221(100.0),177(54.1),109(22.6),149(8.8),123(4.2),99(1.6)	N	33.8	-
A29	8.50	caffeic Acid isomer	$C_9H_8O_4$	179.03498	179.03433	6.5	-3.64	135(100.0),179(23.2),91(5.1),107(1.7),109(1.1),59(0.8),71(0.6)	N	808.9	-
A30	8.64	sibiricose A5	$C_{22}H_{30}O_{14}$	517.15627	517.15625	8.5	-0.06	175(100.0),160(70.2),517(15.6),132(15.0),59(13.9),193(13.8),113(4.58)	N	189.9	-
A31	8.78	6-O-(e)-p-coumaroyl glucopyranose isomer	$C_{15}H_{18}O_8$	325.09289	325.09280	7.5	-0.28	265(100.0),145(93.4),163(72.6),119(69.5),235(42.3),133(13.4)	N	409.7	-

A32	9.36	sibiricose A1 isomer	$C_{23}H_{32}O_{15}$	547.16684	547.16687	8.5	0.05	205(100.0),190(62.6),175(23.3),547(16.4),223(10.2),149(3.6),367(3.1),119(2.5)	N	39.4	424.2
A33	9.46	5-hydroxyferulic acid isomer	$C_{10}H_{10}O_5$	209.04554	209.04495	6.5	-2.85	150(100.0),194(80.8),209(63.7),153(19.5),165(15.9),137(6.4),149(5.7),151(4.68),195(4.67)	N	191.0	-
A34	9.87	6-O-(e)-p-coumaroyl glucopyranose isomer	$C_{15}H_{18}O_8$	325.09289	325.09274	7.5	-0.46	145(100.0),265(95.9),163(79.0),119(72.3),205(52.6),235(36.0)133(15.3),325(8.4)	N	180.3	-
A35	10.03	gluconapoleiferin isomer	$C_{12}H_{21}NO_{10}S_2$	402.05341	402.05002	3.5	-4.43	97(100.0),402(64.7),75(62.4),80(19.4),358(5.7),316(5.8),284(3.2)	N	152.7	-
A36	10.22	sinapine	$C_{16}H_{24}NO_5$	310.16489	310.16437	5.5	-1.71	251(100.0),175(23.3),207(14.0),147(12.3),119(12.3),310(8.3),91(5.8)	P	132958.0	164631.9
A37	10.30	ferulic acid isomer	$C_{10}H_{10}O_4$	193.05063	193.05005	6.5	-3.02	134(100.0),193(20.7),178(17.4),149(9.8),58(7.1),117(6.4),135(5.1),89(3.4)	N	168.0	-
A38	10.31	4-methoxy-3-indolylmethyl glucosinolate isomer	$C_{17}H_{22}N_2O_{10}S_2$	477.06430	477.06442	8.5	2.53	97(100.0),477(58.2),75(33.8),80(18.5),259(2.2),59(1.7),175(1.3),89(1.3)	N	209.8	-
A39	10.31	3,4,5,6-tetrahydroxyoxan-2-yl]methyl (E)-3-(4-hydroxy-3-methoxyphenyl)prop-2-enoate isomer	$C_{16}H_{20}O_9$	355.10345	355.10345	7.5	-0.02	193(100.0),295(85.8),235(77.7),175(76.3),191(39.6),265(38.0),355(6.0)	N	913.1	-
A40	10.62	caffeic acid hexoside isomer	$C_{15}H_{18}O_9$	341.08780	341.08768	7.5	-0.37	137(100.0),93(83.8),59(50.5),71(7.6),281(7.1),341(6.1),161(4.5),101(3.8)	N	163.0	-
A41	10.90	6-O-(e)-p-coumaroyl glucopyranose isomer	$C_{15}H_{18}O_8$	325.09289	325.09265	7.5	-0.74	265(100.0),205(74.9),325(62.3),223(51.3),175(30.7),149(27.4),163(18.1)	N	85.5	-
A42	10.93	3,4,5,6-tetrahydroxyoxan-2-yl]methyl (E)-3-(4-hydroxy-3,5-dimethoxyphenyl)prop-2-enoate isomer	$C_{17}H_{22}O_{10}$	385.11402	385.11404	7.5	0.05	325(100.0),223(87.7),265(79.3),205(72.8),149(52.7),295(44.0),385(21.2)	N	2009.5	-

A43	11.07	4-methylpentyl glucosinolate isomer	$C_{13}H_{25}NO_9S_2$	402.08979	402.09000	2.5	3.24	97(100.0),402(64.7),95(62.4),80(19.4),358(5.7),316(5.6),87(3.1)	N	50.6	844.7
A44	11.17	sibiricose A5 isomer	$C_{22}H_{30}O_{14}$	517.15627	517.15662	8.5	0.66	175(100.0),193(79.3),160(65.7),517(65.1),235(18.5),149(12.1),295(7.7),265(7.5)	N	70.6	-
A45	11.21	sibiricose A1 isomer	$C_{23}H_{32}O_{15}$	547.16684	547.16730	8.5	0.84	205(100.0),190(64.3),175(24.5),367(23.4),223(20.8),547(20.5),149(11.4),337(5.9)	N	940.5	-
A46	11.28	3,4,5,6-tetrahydroxyoxan-2-yl]methyl (E)-3-(4-hydroxy-3-methoxyphenyl)prop-2-enoate isomer	$C_{16}H_{20}O_9$	355.10345	355.10349	7.5	0.10	295(100.0),134(97.7),193(95.4),235(86.6),175(78.5),265(54.3),355(2.8)	N	1137.0	-
A47	11.45	chlorogenic acid hemihydrate isomer	$C_{16}H_{20}O_{10}$	371.09837	371.09830	7.5	-0.19	223(100.0),149(77.5),121(9.5),371(7.7),129(5.6),179(4.2)	N	157.7	-
A48	11.52	sibiricose A1	$C_{23}H_{32}O_{15}$	547.16684	547.16711	8.5	0.49	205(100.0),223(97.7),547(76.6),190(62.5),149(28.7),175(25.1),265(21.5),325(9.9),295(9.4)	N	50.6	844.7
A49	11.66	4-methylpentyl glucosinolate iosmer	$C_{13}H_{25}NO_9S_2$	402.08979	402.08984	2.5	0.11	97(100.0),402(79.2),75(60.8),80(16.7),59(2.5),85(2.2),259(1.9)	N	47.7	1038.8
A50	11.66	P-coumaric acid isomer	$C_9H_8O_3$	163.04006	163.03928	6.5	-4.83	119(100.0),163(9.2),85(3.1),59(2.3),93(1.0),71(0.9)	N	1146.4	-
A51	11.80	6-O-(e)-p-coumaroyl glucopyranose isomer	$C_{15}H_{18}O_8$	325.09289	325.09271	7.5	-0.56	265(100.0),205(89.2),325(72.8),223(56.2),221(51.8),175(30.2)	N	170.0	-
A52	11.80	3,4,5,6-tetrahydroxyoxan-2-yl]methyl (E)-3-(4-hydroxy-3,5-dimethoxyphenyl)prop-2-enoate isomer	$C_{17}H_{22}O_{10}$	385.11402	385.11401	7.5	-0.03	325(100.0),223(88.7),265(82.4),295(53.7),205(49.6),149(43.8),221(22.8),179(14.0),385(6.8)	N	2722.3	-

A53	11.92	sulforaphene	$C_6H_9NOS_2$	176.01983	176.01965	2.5	-1.03	112(100.0),176(77.3),96(73.1),85(53.1),87(42.9),53(38.3),103(34.6),117(15.4)	P	433526.2	-
A54	11.96	allyl Isothiocyanate isomer	C_4H_5NS	100.02154	100.02185	2.5	3.04	100(100.0),67(54.7),55(38.9),61(26.6),86(4.5),85(3.1)	P	210.9	-
A55	11.98	gluconapoleiferin isomer	$C_{12}H_{21}NO_{10}S_2$	402.05341	402.05014	3.5	-4.13	97(100.0),402(17.1),75(13.7),153(6.9),80(4.6),181(2.0),116(1.7)	N	149.6	-
A56	11.98	caffeic acid hexoside isomer	$C_{15}H_{18}O_9$	341.08780	341.08771	7.5	-0.28	137(100.0),93(78.8),59(49.4),71(11.9),341(11.7),101(5.8),281(5.5),113(5.0)	N	4135.8	-
A57	12.04	caffeic Acid isomer	$C_9H_8O_4$	179.03498	179.03442	6.5	-3.14	109(100.0),179(64.4),135(4.6),123(2.9),91(2.7),95(0.8),71(0.5)	N	505.4	-
A58	12.10	but-3-enyl isothiocyanate isomer	C_5H_7NS	114.03719	114.03744	2.5	2.14	72(100.0),114(49.8),55(25.1),71(1.8),81(0.8),53(0.7),97(0.5),85(0.4)	P	10054.8	-
A59	12.13	1-(methylsulfinyl)but-1-ene isomer	$C_5H_{10}OS$	119.05251	119.05271	0.5	1.66	119(100.0),55(99.6),65(18.2),91(5.8),87(2.5)	P	1372.2	-
A60	12.74	3,4,5,6-tetrahydroxyoxan-2-yl]methyl (E)-3-(4-hydroxy-3,5-dimethoxyphenyl)prop-2-enoate isomer	$C_{17}H_{22}O_{10}$	385.11402	385.11404	7.5	0.05	267(100.0),385(57.4),249(38.4),113(36.5),223(16.3),149(12.5),135(11.8),175(2.8)	N	465.1	-
A61	13.37	ferulic acid isomer	$C_{10}H_{10}O_4$	193.05063	193.05003	6.5	-3.12	134(100.0),178(70.2),193(38.9),149(27.7),137(20.2),179(4.7),121(3.1)	N	170.6	-
A62	13.67	sinapic acid	$C_{11}H_{12}O_5$	223.06119	223.06071	6.5	-2.18	208(100.0),223(61.8),193(60.6),149(55.5),164(41.8),163(15.7),179(12.7),121(12.2)	N	17.6	800.8

A63	13.70	{[(e)-[(4E)-5-methanesulfinyl-1-{[3,4,5-trihydroxy-6-({[(2E)-3-(4-hydroxy-3,5-dimethoxyphenyl)prop-2-enoyl]oxy}methyl)oxan-2-yl]sulfanyl}pent-4-en-1-ylidene]amino]oxy}sulfonic acid isomer	$C_{23}H_{31}NO_{14}S_3$	640.08338	640.08356	9.5	0.27	97(100.0),640(34.2),80(16.4),149(13.4),223(10.5),164(9.7),625(2.5),205(1.5),147(1.4),325(1.3)	N	1837.1	8047.1
A64	14.40	raphasatin isomer	$C_6H_9NS_2$	160.02491	160.02492	2.5	0.02	160(100.0),112(52.4),68(14.6),80(11.9),126(8.9),101(8.9),127(7.8),113(7.0),85(3.5)	P	7687.8	-
A65	14.48	caffeic acid hexoside isomer	$C_{15}H_{18}O_9$	341.08780	341.08765	7.5	-0.46	137(100.0),93(74.9),59(2.1),341(0.5),65(0.3),85(0.3)	N	81.5	428.4
A66	14.76	ferulic acid isomer	$C_{10}H_{10}O_4$	193.05063	193.05005	6.5	-3.02	134(100.0),178(39.4),93(36.0),193(27.5),149(17.0),121(11.2),137(11.0),165(9.0)	N	493.2	-
A67	14.91	sinapic acid isomer	$C_{11}H_{12}O_5$	223.06119	223.06073	6.5	-2.09	208(100.0),149(83.6),223(74.8),193(62.9),164(60.7),121(26.3),179(14.8),163(14.8),135(11.17)	N	41.7	2000.3
A68	16.21	[3,4,5-trihydroxy-6-[[(E)-3-(4-hydroxyphenyl)prop-2-enoyl]oxymethyl]oxan-2-yl] 3,4,5-trihydroxybenzoate isomer	$C_{22}H_{22}O_{12}$	477.10384	477.10397	12.5	0.25	477(100.0),314(45.5),271(32.8),243(32.0),285(22.3),315(8.5),299(7.6)	N	53.5	-
A69	16.31	3,6′-disinapoylsucrose	$C_{34}H_{42}O_{19}$	753.22475	753.22479	14.5	0.05	205(100.0),190(81.6),175(29.0),223(12.1),547(11.3),149(8.9),753(6.3),265(3.4)	N	514.3	4723.4
A70	16.33	alyssin isomer	$C_7H_{13}NOS_2$	192.05113	192.05099	1.5	-0.74	69(100.0),128(69.0),133(15.22),192(12.1),72(0.2),65(3.4),53(1.9)	P	6587.3	-
A71	16.34	4-pentenyl isothiocyanate isomer	C_6H_9NS	128.05284	128.05292	2.5	0.57	69(100.0),128(60.4),72(29.7),86(17.6),112(11.1),67(3.4),53(3.0)	P	648.3	-

A72	16.59	3′-O-sinapoyl-6-O-feruloylsucrose isomer	$C_{33}H_{40}O_{18}$	723.21418	723.21448	14.5	0.41	175(100.0),160(80.44),205(64.9),190(50.5),132(20.3),175(20.1),723(14.5),547(12.4)	N	53.1	1246.7
A73	16.84	helonioside A isomer	$C_{32}H_{38}O_{17}$	693.20362	693.20386	14.5	0.34	175(100.0),160(76.6),193(13.3),517(11.5),693(10.4),499(5.4),235(3.9),337(3.2)	N	147.1	-
A74	17.44	6-sinapoylglucoraphenin isomer	$C_{23}H_{31}NO_{13}S_3$	624.08847	624.08893	9.5	0.46	97(100.0),96(52.4),624(36.1),149(19.1),80(16.7),223(16.5),164(14.7),121(2.5),325(1.6)	N	21.8	80.8
A75	17.47	g(8-O-4)FA sulfate isomer	$C_{20}H_{22}O_{11}S$	469.08100	469.08109	10.5	0.18	97(100.0),469(51.0),241(28.6),227(5.4),80(3.8)	N	30.8	-
A76	17.72	6-sinapoylglucoraphenin isomer	$C_{23}H_{31}NO_{13}S_3$	624.08847	624.08893	9.5	0.46	97(100.0),67(56.8),96(50.6),624(39.3),149(19.8),164(18.7),223(18.4),80(15.6)	N	19.8	446.8

Note: S: raw RS; C: stir-fried RS; -: no detected.

Table S2. Metabolites *in vivo* of raw and stir-fried RS decoctions.

No.	tR (min)	Compound	Formula	Theoretical (m/z)	Experiment (m/z)	RDB	error (ppm)	Fragment ions	ion mode	X	N	F	G
M1	0.97	glucoraphasatin glucosylation+demethylation isomer	$C_{17}H_{29}NO_{14}S_3$	566.06773	566.06769	4.5	-0.09	97(100.0),566(60.2),96(59.8),75(24.3),259(2.8),291(2.7),81(1.6),324(1.5),85(1.3)	N			C	
M2	1.00	glucoraphenin	$C_{12}H_{21}NO_{10}S_3$	434.02548	434.02554	3.5	0.14	97(100.0),96(62.6),434(62.4),75(30.0),80(12.5),419(4.7),259(3.2)	N	C	C	S,C	
M3	1.05	sinapine sulfation+dihydroxylation isomer	$C_{16}H_{26}NO_8S$	392.13736	392.13712	4.5	-0.62	251(100.0),310(45.4),175(19.6),87(19.2),392(16.2),207(13.3),328(10.10.1),147(7.7),119(6.5),123(6.1)	P			S,C	
M4	1.26	sulforaphene glucosylation isomer	$C_{12}H_{19}NO_6S_2$	338.07265	338.07245	3.5	-0.61	176(100.0),128(71.7),146(39.9),61(25.8),71(21.1),160(19.9),85(12.3),338(10.2),111(9.4)	P			S,C	
M5	1.33	sinapine hydration+cysteinylation isomer	$C_{19}H_{31}N_2O_7S$	431.18464	431.18448	5.5	-0.39	251(100.0),431(75.7),286(27.3),175(17.8),167(13.5),182(12.9),207(12.7),132(11.9),70(9.4)	P			S,C	
M6	1.43	sinapine hydration isomer	$C_{16}H_{26}NO_6$	328.17546	328.17523	4.5	-0.71	87(100.0),328(48.2),123(26.6),183(23.1),95(16.1),155(15.1),70(7.1),195(5.4),134(4.0)	P			S,C	
M7	1.47	5-(methylsulfinyl)-4-pentenenitrile demethylation+glucosylation isomer	$C_{11}H_{17}NO_6S$	292.08493	292.08463	3.5	-1.04	114(100.0),215(89.5),292(87.3),71(71.8),86(59.0),233(55.8),130(47.7),69(33.9),97(33.4)	P			C	

M8	1.59	glucoraphasatin demethylation isomer	$C_{11}H_{19}NO_9S_3$	404.01491	404.01498	3.5	0.16	113(100.0),69(66.1),97(23.4),404(9.9),80(6.7),108(4.9)	N			C	
M9	1.74	5-(methylsulfinyl)-4-pentenenitrile demethylation+dihydroxylation isomer	C_5H_9NOS	132.04776	132.04778	1.5	0.14	86(100.0),132(76.8),71(52.9),114(23.4),81(19.6),57(9.1),55(8.3),87(4.6),69(3.9),97(3.0)	P			S,C	
M10	1.78	sinapine sulfation+dihydroxylation isomer	$C_{16}H_{26}NO_8S$	392.13736	392.13687	4.5	-1.26	251(100.0),310(43.8),87(19.0),175(17.1),392(16.1),207(11.7),328(9.7),147(72),119(5.6),123(5.5),179(5.3)	P			S,C	
M11	1.84	5-(methylsulfinyl)-4-pentenenitrile cysteinylation isomer	$C_9H_{14}N_2O_2S_2$	247.05694	247.05679	3.5	-0.63	247(100.0),101(59.5),70(34.3),55(23.7),113(12.6),159(7.5),61(3.4),199(2.6),153(2.3)201(2.2)	P			S,C	
M12	1.87	5-(methylsulfinyl)-4-pentenenitrile isomer	C_6H_9NOS	144.04776	144.04765	2.5	-0.77	87(100.0),144(78.7),104(27.6),53(21.7),89(12.5),65(7.5),127(5.5)	P	S	S,C	S,C	C
M13	2.11	glucoraphenin-oxidation +dihydroxylation+demethylation isomer	$C_{11}H_{21}NO_9S_3$	406.03056	406.03033	2.5	-0.58	97(100.0),406(74.2),75(48.6),96(45.1),80(15.0),115(8.4),164(3.1),75(2.9),59(2.3)	N			C	
M14	2.14	glucoraphasatin glucosylation isomer	$C_{18}H_{31}NO_{14}S_3$	580.08338	580.08417	4.5	1.35	97(100.0),580(75.8),96(26.9),68(12.2),80(11.1),221(7.4),75(6.2),101(5.8),59(5.7)	N			C	
M15	2.15	6-[(methylsulfinyl)methyl]-1,3-thiazinan-2-thione isomer	$C_6H_{11}NOS_3$	210.00755	210.0074	1.5	-0.73	146(100.0),87(73.5),118(9.9),72(5.1),85(3.6),210(1.5),53(1.2),112(1.0)	P	S,C	S,C	S,C	

M16	2.30	sinapine hydration isomer	$C_{16}H_{26}NO_6$	328.17546	328.17545	4.5	-0.04	87(100.0),328(47.1),123(27.4),183(24.4),95(18.1),155(162),140(6.1),70(4.1),134(2.8),87(2.5),165(2.4),195(2.2)	P			S
M17	2.52	4-hydroxyglucobrassicin isomer	$C_{16}H_{20}N_2O_{10}S_2$	463.04865	463.0488	8.5	0.30	97(100.0),96(24.0),75(23.1),463(17.9),169(5.7),80(4.6),285(3.2)	N			S
M18	2.54	sulforaphene dihydroxylation+glycination isomer	$C_8H_{14}N_2O_2S_2$	235.05694	235.05692	2.5	-0.11	74(100.0),235(47.3),155(5.7),138(5.2),139(3.1),75(3.0),111(2.8),121(1.9)	P		S,C	
M19	2.61	5-(methylsulfinyl)-4-pentenenitrile cysteinylation isomer	$C_9H_{14}N_2O_2S_2$	247.05694	247.05682	3.5	-0.51	247(100.0),101(55.0),55(24.1),113(12.1),159(7.6),75(6.4),86(4.1),70(3.3),61(2.9),201(2.5)	P			S,C
M20	2.62	6-[(methylsulfinyl)methyl]-1,3-thiazinan-2-thione hydroxymethylation isomer	$C_7H_{13}NO_2S_3$	240.01811	240.01791	1.5	-0.86	240(100.0),87(81.5),132(146.0),91(17.7),167(7.8),160(7.7),133(5.1),85(2.4),69(1.4)	P	S,C		
M21	2.80	glucoraphasatin glucosylation isomer	$C_{18}H_{31}NO_{14}S_3$	580.08338	580.0838	4.5	0.71	97(100.0),580(55.6),96(31.8),80(10.5),59(7.5),221(7.4),95(6.9),101(6.9),71(6.9)	N			C
M22	3.19	4-methylthiobutyl glucosinolate isomer	$C_{12}H_{23}NO_9S_3$	420.04621	420.04648	2.5	0.63	97(100.0),420(67.4),96(48.4),75(47.8),80(12.6),259(4.9),71(4.9)	N			S,C
M23	3.44	glucoraphasatin isomer	$C_{12}H_{21}NO_9S_3$	418.03056	418.03064	3.5	0.18	97(100.0),96(54.0),75(48.2),418(42.2),80(16.0),259(2.6),176(2.4),419(2.0)	N			C

M24	3.63	sulforaphene glucosylation+dihydroxylation isomer	$C_{12}H_{21}NO_6S_2$	340.0883	340.08813	2.5	-0.52	130(100.0),178(28.2),85(7.5),71(5.5),131(2.5),97(2.4),145(2.3),127(1.5),69(1.5)	P			C
M25	3.65	glucoraphasatin sulfation isomer	$C_{12}H_{21}NO_{12}S_4$	497.98738	497.98737	3.5	-0.02	97(100.0),418(68.8),176(35.2),241(24.4),259(16.9),75(15.8),96(14.8),275(5.9),80(5.6),419(5.2)	N			C
M26	3.68	5-(methylsulfinyl)-pentanenitrile methylation isomer	$C_7H_{13}ONS$	160.07906	160.07899	1.5	-0.45	132(100.0),113(76.4),96(27.7),150(26.2),69(15.0),160(11.6)	P	S,C	S,C	
M27	3.99	Sulforaphene dihydroxylation+glycination isomer	$C_8H_{14}N_2O_2S_2$	235.05694	235.05672	2.5	-0.96	74(100.0),235(44.1),138(5.7),155(5.6),128(4.0),75(3.6),94(2.8),121(1.9)	P		S,C	
M28	4.73	glucoraphasatin isomer	$C_{12}H_{21}NO_9S_3$	418.03056	418.03058	3.5	0.03	97(100.0),418(54.5),96(49.6),75(40.4),80(14.3),176(2.7),259(2.5)	N		S,C	S,C
M29	4.89	4-hydroxyphenyllactic acid isomer	$C_9H_{10}O_4$	181.05063	181.04997	5.5	2.40	163(100.0),181(96.4),135(84.3),119(54.3),73(	N	S	S	
M30	5.05	sinapic acid glucuronidation isomer	$C_{17}H_{20}O_{11}$	399.09328	399.09335	8.5	0.16	223(100.0),208(49.9),113(46.7),193(34.5),85(33.6),149(31.9),164(21.4),71(17.7),59(15.3),175(9.5),399(1.9)	N		S,C	
M31	5.34	sinapine glucuronidation isomer	$C_{22}H_{32}NO_{11}$	486.19698	486.19684	7.5	-0.30	251(100.0),175(13.2),486(12.6),207(8.6),310(6.6),147(5.6),119(4.6),91(1.5),427(1.5)	P		S,C	

M32	5.75	sinapic acid glucuronidation isomer	$C_{17}H_{20}O_{11}$	399.09328	399.09311	8.5	-0.44	223(100.0),193(40.7),208(39.7),85(31.5),113(31.3),59(40.0),149(24.4),71(21.1)	N	S,C			
M33	5.78	sulforaphene dihydroxylation+hydroxymethylation isomer	$C_7H_{13}NO_2S_2$	208.04604	208.04599	1.5	-0.27	160(100.0),117(70.9),87(32.7),96(13.2),53(11.4),85(7.6),61(7.5),208(1.3)	P		S,C		
M34	5.97	5-(methylsulfinyl)-4-pentenenitrile acetylation+deoxidation+dihydroxylation isomer	$C_8H_{13}NOS$	172.07906	172.07901	2.5	-0.30	87(100.0),172(64.7),124(43.7),85(4.0),59(3.5),126(2.7),53(2.5),81(1.30	P			C	
M35	6.15	sinapic acid sulfation isomer	$C_{11}H_{12}O_8S$	303.01801	303.01782	6.5	-0.63	223(100.0),208(39.6),164(13.6),193(12.2),303(11.8),149(11.6),97(10.2),179(6.8),80(5.6)	N		S		
M36	6.27	5-(methylsulfinyl)-4-pentenenitrile deoxidation isomer	C_6H_9NS	128.05284	128.05293	2.5	0.65	87(100.0),61(10.8),53(7.6),59(6.2),85(5.3),128(3.4)	P	S,C	S,C	S,C	C
M37	6.30	sulforaphene methylation+dehydrogenation isomer	$C_7H_{13}NOS_2$	192.05113	192.05104	1.5	-0.48	87(100.0),128(57.9),146(13.2),192(6.5),164(5.8),69(5.5),85(3.4)	P		S,C		
M38	6.39	sinapic acid sulfation+dihydroxylation isomer	$C_{11}H_{14}O_8S$	305.03366	305.03351	5.5	-0.50	225(100.0),305(83.0),80(73.7),59(40.6),165(27.6),81(8.5),181(7.8),153(3.3)	N		S,C		
M39	6.44	sinapic acid Sulfation isomer	$C_{11}H_{12}O_8S$	303.01801	303.01807	6.5	0.19	223(100.0),208(56.2),68(21.0),193(19.2),149(18.7),164(18.2),97(10.0),179(9.2),80(7.2),135(6.4),303(5.5)	N	S,C		S,C	

M40	6.44	sulforaphene methylation isomer	$C_7H_{11}NOS_2$	190.03548	190.03551	2.5	0.15	162(100.0),190(45.7),126(29.1),65(8.2),114(4.4),116(2.2),163(1.3),131(0.9),144(0.7),89(0.6),	P		S,C	
M41	6.49	sinapine demethylation isomer	$C_{15}H_{22}NO_5$	296.14924	296.14911	5.5	-0.47	237(100.0),161(21.1),133(18.4),193(16.3),296(12.7),151(1.8),178(1.5),165(1.5),175(1.2)	P			S,C
M42	6.82	sulforaphene glycination isomer	$C_8H_{12}N_2O_2S_2$	233.04129	233.0414	3.5	0.45	169(100.0),117(72.8),112(40.4),215(34.9),141(30.9),85(19.7),53(13.2),70(12.4),233(3.6)	P		S,C	
M43	6.87	sinapic acid sulfation+dihydroxylation isomer	$C_{11}H_{14}O_8S$	305.03366	305.03348	5.5	-0.59	225(100.0),305(99.1),80(74.4),59(36.7),165(28.3),81(8.8),151(6.9),181(5.1),153(2.6)	N		S,C	
M44	7.25	sinapic acid sulfation+dihydroxylation isomer	$C_{11}H_{14}O_8S$	305.03366	305.03348	5.5	-0.59	225(100.0),305(86.4),80(72.3),59(35.2),165(28.0),81(8.4),181(7.7),151(6.9),153(2.6)	N		S,C	
M45	7.42	sinapic acid glucuronidation+deoxidation isomer	$C_{17}H_{20}O_{10}$	383.09837	383.09814	8.5	-0.60	207(100.0),163(79.7),113(79.1),383(53.4),85(36.4),175(32.3),71(21.2),59(19.7)	N		S,C	
M46	7.49	sinapic acid sulfation isomer	$C_{11}H_{12}O_8S$	303.01801	303.01791	6.5	-0.33	223(100.0),208(42.8),164(15.1),193(13.5),149(12.0),303(11.4),97(10.4),179(7.2),80(6.5),163(3.7)	N		S,C	
M47	7.64	sinapic acid glucuronidation isomer	$C_{17}H_{20}O_{11}$	399.09328	399.09329	8.5	0.01	223(100.0),208(57.6),113(52.0),85(33.1),193(23.2),149(23.0),179(13.9),71(13.6)	N	S,C	S,C	

M48	7.64	1-(methylsulfinyl)but-1-ene acetylation isomer	$C_7H_{12}O_2S$	161.06307	161.0631	1.5	0.14	144(100.0),71(6.8),61(5.7),116(5.1),117(4.3),134(4.0),85(3.0),161(1.9)	P		S,C	
M49	7.69	caffeic acid hexoside isomer	$C_{15}H_{18}O_9$	341.0878	341.08759	7.5	-0.63	341(100.0),113(67.0),121(42.4),165(42.2),85(38.9),59(31.6)	N		S	
M50	7.70	5-(Methylsulfinyl)-4-pentenenitrile cysteinylation isomer isomer	C9H14N2O2 S2	247.05694	247.05688	3.5	-0.27	199(100.0),153(42.2),101(24.8),55(14.1),247(10.4)	P		C	
M51	7.97	4-coumaroylcholine isomer	$C_{14}H_{20}NO_3$	250.14377	250.14369	5.5	-0.32	250(100.0),150(70.8),55(33.9),101(23.9),83(13.2),59(8.4)	P			S
M52	7.98	sinapic acid sulfation+dihydroxylation isomer	$C_{11}H_{14}O_8S$	305.03366	305.03378	5.5	0.39	225(100.0),305(84.1),80(64.0),59(38.0),68(30.6),165(26.8),181(7.0)	N	S,C		
M53	8.01	sinapic acid glucuronidation+dihydroxylation isomer	$C_{17}H_{22}O_{11}$	401.10893	401.10886	7.5	-0.19	113(100.0),225(60.1),401(52.7),85(52.2),59(49.7),71(35.2),99(23.8),175(23.5),95(16.2),165(16.1)	N	S,C	S,C	
M54	8.04	sulforaphene methylation isomer	$C_7H_{11}NOS_2$	190.03548	190.03542	2.5	-0.33	162(100.0),97(19.1),107(16.7),142(12.0),190(11.3),145(10.2),173(7.3)	P		C	
M55	8.22	6-[(Methylsulfinyl)methyl]-1,3-thiazinan-2-thione hydroxymethylation isomer	$C_7H_{13}NO_2S_3$	240.01811	240.01807	1.5	-0.20	61(100.0),176(92.1),98(66.8),112(45.0),160(42.5),80(30.5),128(26.7),240(7.1)	P		C	
M56	8.34	sulforaphene hydroxylation isomer	$C_6H_9NO_2S_2$	192.01474	192.01459	2.5	-0.82	85(100.0),133(93.6),146(84.1),192(75.9),69(67.1),73(47.6),61(34.7)	P		S,C	

M57	8.43	glucoberteroin isomer	$C_{13}H_{25}NO_9S_3$	434.06186	434.06195	2.5	0.19	97(100.0),434(79.7),96(50.1),75(43.7),80(13.9),314(13.6),286(4.8),344(4.6)	N		C
M58	8.47	caffeic Acid isomer	$C_9H_8O_4$	179.03498	179.03447	6.5	-2.86	135(100.0),179(23.3),107(2.3),91(1.6)	N		S
M59	8.47	sinapic acid sulfation isomer	$C_{11}H_{12}O_8S$	303.01801	303.01807	6.5	0.19	81(100.0),303(68.4),223(28.9),208(10.6),80(7.4),165(6.4),285(4.9),221(4.3),193(3.7),149(3.5)	N		S,C
M60	8.55	sinapine methylation+hydrogenation isomer	$C_{17}H_{28}NO_5$	326.19619	326.19604	4.5	-0.49	267(100.0),252(15.8),73(14.1),234(12.2),326(8.5),223(7.5),195(7.5),107(6.4),237(6.1)	P		S,C
M61	8.58	sulforaphene acetylcysteineification isomer	$C_{11}H_{16}N_2O_3S_3$	321.03958	321.03934	4.5	-0.75	130(100.0),192(84.7),84(64.3),133(17.1),85(10.8),116(9.4),146(7.8),321(5.1),176(1.5)	P	S,C	
M62	8.61	sulforaphene cysteinylation+dimethylation+dihydroxylation isomer	$C_{11}H_{18}N_2O_4S_3$	339.05014	339.04971	3.5	-1.29	87(100.0),176(91.1),130(78.8),69(72.4),117(41.6),68(39.0),192(32.3),146(11.0),339(2.2)	P	S,C	
M63	8.64	sibiricose A5 isomer	$C_{22}H_{30}O_{14}$	517.15627	517.15668	8.5	0.78	175(100.0),160(56.7),517(15.3),59(11.8),193(10.3),134(8.2),89(7.9),71(6.4)	N		S
M64	8.84	sinapic acid glucuronidation+deoxidation isomer	$C_{17}H_{20}O_{10}$	383.09837	383.09845	8.5	0.21	163(100.0),113(92.9),207(92.3),383(59.0),85(45.5),59(38.0),175(33.4),71(27.3),87(25.1)	N	S,C	

M65	8.90	sinapine demethylation+deoxidation+dihydroxylation isomer	$C_{15}H_{24}NO_4$	282.16998	282.16998	4.5	-0.02	137(100.0),87(37.4),282(34.6),223(18.3),70(14.2),107(9.7),218(9.7),87(5.2),117(5.1)	P			S,C
M66	9.03	5-(Methylsulfinyl)-4-pentenenitrile deoxidation+glycination isomer	$C_8H_{12}N_2OS$	185.07431	185.07425	3.5	-0.33	185(100.0),114(42.2),157(38.7),126(36.0),149(20.1),55(17.2),71(16.5),121(14.3)	P		S,C	
M67	9.11	sinapic acid sulfation+deoxidation isomer	$C_{11}H_{12}O_7S$	287.02309	287.02298	6.5	-0.41	207(100.0),287(75.3),163(53.4),122(11.4),80(5.0),109(2.0),137(1.5),269(1.1)	N		S,C	
M68	9.22	sinapine demethylation+deoxidation isomer	$C_{15}H_{22}NO_4$	280.15433	280.15408	5.5	-0.91	221(100.0),177(25.9),145(25.2),280(10.3),117(4.4),206(4.3),149(1.9),178(1.4),89(0.6)	P			S,C
M69	9.29	sinapic acid glucuronidation+dihydroxylation isomer	$C_{17}H_{22}O_{11}$	401.10893	401.10889	7.5	-0.11	113(100.0),225(54.6),401(49.7),85(45.9),59(43.2),71(25.6),175(23.2),99(19.9),95(15.3),165(13.4)	N	S	S,C	
M70	9.29	sinapine dihydroxylation	$C_{16}H_{26}NO_5$	312.18054	312.1799	4.5	-2.08	167(100.0),312(35.0),87(34.5),253(12.2),168(6.7),123(1.8),88(1.1),60(0.7),99(0.4)	P			S,C
M71	9.34	sibiricose A1 isomer	$C_{23}H_{32}O_{15}$	547.16684	547.16718	8.5	2.62	205(100.0),190(49.9),175(18.3),547(17.5),59(10.7),223(10.6),89(7.4)	N			S,C
M72	9.48	sulforaphene cysteinylation+methylation isomer	$C_{10}H_{16}N_2O_2S_3$	293.04466	293.04446	3.5	-0.70	245(100.0),199(71.9),117(8.0),153(5.8),170(3.1),87(3.0),151(2.8),76(2.6),184(2.6),293(0.9)	P		S,C	

M73	9.53	sinapic acid sulfation+glycination isomer	$C_{13}H_{15}NO_9S$	360.03947	360.03943	7.5	-0.13	360(100.0),280(50.8),221(39.5),178(38.2),193(37.7),206(34.9),80(9.9),149(7.7)	N		S,C		
M74	9.82	sulforaphene acetylcysteineification isomer	$C_{11}H_{16}N_2O_3S_3$	321.03958	321.03943	4.5	-0.47	130(100.0),192(84.2),84(64.6),133(17.9),56(15.7),85(11.5),321(5.2),176(0.4)	P		S,C	S	
M75	9.82	sulforaphene cysteinylation isomer	$C_9H_{14}N_2O_2S_3$	279.02901	279.02872	3.5	-1.06	149(100.0),231(95.6),221(79.7),185(79.6),147(69.2),133(53.4),113(44.8),159(44.3),279(34.1),176(1.4)	P		S,C		
M76	9.86	sulforaphene cysteinylation+dimethylation+dihydroxylation isomer	$C_{11}H_{18}N_2O_4S_3$	339.05014	339.04984	3.5	-0.90	176(100.0),87(80.7),130(78.9),116(67.3),69(66.5),192(25.6),68(32.9),85(16.1),146(11.7),339(3.0)	P		S,C		
M77	11.02	4-methylpentyl glucosinolate isomer	$C_{13}H_{25}NO_9S_2$	402.08979	402.09	2.5	0.51	97(100.0),402(81.8),75(61.8),96(50.2),80(22.3),87(16.1),146(4.2)	N			S,C	
M78	10.06	sulforaphene methylation isomer	$C_7H_{11}NOS_2$	190.03548	190.03549	2.5	0.04	82(100),83(82.1),167(60.4),190(41.5),149(37.8),67(32.8),133(20.1)	P	S			
M79	10.20	sulforaphene glucosylation+deoxidation isomer	$C_{12}H_{19}NO_5S_2$	322.07774	322.07742	3.5	-1.00	160(100.0),322(17.1),112(12.4),202(9.4),85(8.9),304(4.9),161(3.1),130(2.5),69(2.4)	P			C	
M80	10.22	sinapine	$C_{16}H_{24}NO_5$	310.16489	310.16495	5.5	0.16	251(100.0),175(23.0),207(10.6),310(9.9),147(9.5),119(8.8)	P	S,C	S,C	S,C	S,C

M81	10.63	sinapic acid deoxidation isomer	$C_{11}H_{12}O_4$	207.06628	207.06573	6.5	-2.67	163(100.0),207(89.8),122(18.4),164(16.0),208(15.8),109(5.3),137(4.4),59(2.9)	N		S,C	
M82	10.63	sinapic acid glucuronidation+dihydroxylation isomer	$C_{17}H_{22}O_{11}$	401.10893	401.10904	7.5	0.26	113(100.0),401(80.1),225(66.2),85(63.5),59(40.6),71(40.1),151(29.3),99(28.7),165(26.8),175(21.9)	N		S,C	
M83	10.83	sinapic acid sulfation+dihydroxylation isomer	$C_{11}H_{14}O_8S$	305.03366	305.03369	5.5	0.09	305(100.0),225(66.5),151(34.3),165(28.3),80(25.4),149(24.6)	N	S,C	S	
M84	10.87	sinapic acid sulfation+glycination isomer	$C_{13}H_{15}NO_9S$	360.03947	360.03958	7.5	0.29	360(100.0),280(53.3),193(46.8),206(45.9),178(42.9),221(39.4),236(35.8),163(14.1),163(14.1),80(9.1)	N		S,C	
M85	10.93	sinapoylhexoside isomer	$C_{17}H_{22}O_{10}$	385.11402	385.11423	7.5	0.55	113(100.0),385(87.0),209(49.2),85(44.3),175(30.9),59(23.4),71(20.4),99(19.5),123(17.7),191(15.4)	N		S,C	
M86	11.26	sinapic acid glucuronidation isomer	$C_{17}H_{20}O_{11}$	399.09328	399.09314	8.5	-0.36	113(100.0),208(65.2),85(57.7),223(52.2),193(39.8),71(39.7),59(31.8),399(28.9),95(19.8),175(17.3)	N		S,C	
M87	11.69	4-methylpentyl glucosinolate isomer	$C_{13}H_{25}NO_9S_2$	402.08979	402.08994	2.5	0.36	97(100.0),402(65.6),75(62.5),96(49.2),87(5.6),311(3.1)	N			S,C

M88	11.71	sinapic acid sulfation+deoxidation+dihydroxylation isomer	$C_{11}H_{14}O_7S$	289.03874	289.03867	5.5	-0.27	289(100.0),209(64.7),88(43.3),132(26.0),123(16.8),191(16.6),80(14.2),165(9.4),115(9.2),151(6.0)	N		S		
M89	11.89	glucoraphasatin acetylation isomer	$C_{14}H_{23}NO_{10}S_3$	460.04113	460.0412	4.5	0.15	97(100.0),96(58.2),460(47.0),59(21.0),75(13.0),80(13.0),81(4.9)	N			C	
M90	11.92	sulforaphene	$C_6H_9NOS_2$	176.01983	176.01984	2.5	0.05	112(100.0),176(91.8),96(79.8),85(61.5),87(44.6),103(40.7),53(36.7),61(25.2),148(21.1),117(19.3)	P	S,C	S,C	S,C	S,C
M91	11.95	sinapic acid acetylation isomer	$C_{13}H_{14}O_6$	265.07176	265.07181	7.5	0.18	107(100.0),85(65.9),265(24.4),57(21.2),59(15.5),83(6.9),95(4.3),157(4.0)	N		S,C		
M92	12.07	1-(methylsulfinyl)but-1-ene isomer	$C_5H_{10}OS$	119.05251	119.0527	0.5	1.58	91(100.0),103(3.7),79(1.9),60(1.6),65(1.4),119(0.9)	P		S,C	C	
M93	12.08	sinapic acid methylation isomer	$C_{12}H_{14}O_5$	237.07684	237.07651	6.5	-1.42	237(100.0),193(16.4),145(15.8),149(15.3),121(13.9),175(11.1),19(10.8)	N			S,C	
M94	12.10	sulforaphene dihydroxylation	$C_6H_{11}NOS_2$	178.03548	178.03542	1.5	-0.35	114(100.0),72(48.1),55(34.6),119(14.2),178(12.7),85(11.3),91(8.9)	P		S,C		
M95	12.10	but-3-enyl isothiocyanate isomer	C_5H_7NS	114.03719	114.03743	2.5	2.05	72(100.0),114(54.6),55(26.2),86(10.4),69(5.3),79(1.8)	P		S	C	

M96	12.22	sinapic acid methylation isomer	$C_{12}H_{14}O_5$	237.07684	237.07652	6.5	-1.38	145(100.0),237(71.8),119(54.5),117(26.8),163(20.3),149(5.0),59(4.9),137(4.3),193(4.0)	N			S,C
M97	12.35	sinapine hydroxymethylation isomer	$C_{17}H_{26}NO_6$	340.17546	340.17548	5.5	0.05	85(100.0),340(46.7),133(20.2),105(19.8),322(13.9),114(13.2),96(12.5),281(10.1),209(9.4),79(6.0)	P		S,C	
M98	12.59	raphasatin isomer	$C_6H_9NS_2$	160.02491	160.02492	2.5	0.02	112(100.0),160(34.2),87(32.6),85(30.1),53(16.7),61(11.8),127(8.6)	P		S,C	
M99	12.80	glucoraphasatin acetylation isomer	$C_{14}H_{23}NO_{10}S_3$	460.04113	460.04129	4.5	0.35	97(100.0),96(56.8),460(41.1),59(23.6),75(18.0),80(14.3),81(3.6),176(1.6),85(1.5),279(1.3)	N			C
M100	13.04	sinapine hydroxymethylation isomer	$C_{17}H_{26}NO_6$	340.17546	340.17545	5.5	-0.04	85(100.0),340(80.2),281(31.1),105(27.2),133(21.9),79(0.2),166(8.4)	P		S,C	
M101	13.05	sinapic acid glycination isomer	$C_{13}H_{15}NO_6$	280.08266	280.0827	7.5	0.14	178(100.0),280(56.1),193(55.9),206(53.5),109(45.1),221(35.1),149(32.5)	N		S,C	
M102	13.12	sinapic acid dihydroxylation isomer	$C_{11}H_{14}O_5$	225.07684	225.07637	5.5	-2.12	151(100.0),225(88.3),59(49.1),165(25.1),123(21.1),225(13.7),149(6.6),181(5.3)	N		S,C	S,C
M103	13.64	sinapic acid	$C_{11}H_{12}O_5$	223.06119	223.06071	6.5	-2.18	208(100.0),223(80.2),193(68.1),149(53.7),164(33.4),121(22.1),163(19.5),179(15.1),165(13.6)	N	C	S,C	S,C

M104	13.75	{[(e)-[(4E)-5-methanesulfinyl-1-{[3,4,5-trihydroxy-6-({[(2E)-3-(4-hydroxy-3,5-dimethoxyphenyl)prop-2-enoyl]oxy}methyl)oxan-2-yl]sulfanyl}pent-4-en-1-ylidene]amino]oxy}sulfonic acid isomer	$C_{23}H_{31}NO_{14}S_3$	640.08338	640.08356	9.5	0.27	97(100.0),640(34.2),80(16.4),149(13.4),223(10.5),164(9.7),625(2.5),205(1.5),147(1.4),325(1.3)	N		S,C	C
M105	13.95	sinapic acid deoxidation+dihydroxylation isomer	$C_{11}H_{14}O_4$	209.08193	209.08138	5.5	2.61	209(100.0),123(37.0),191(33.3),165(20.4),80(17.6),91(13.2),147(12.0),210(7.4)	N		S,C	S
M106	14.36	raphasatin isomer	$C_6H_9NS_2$	160.02491	160.02493	2.5	0.08	160(100.0),112(61.5),68(15.9),80(11.5),61(11.0),130(10.9),126(8.6),101(8.4)	P			S,C
M107	14.37	glucoraphenin hydroxymethylation+hydroxylation isomer	$C_{13}H_{23}NO_{12}S_3$	480.03096	480.03186	3.5	1.87	206(100.0),252(95.7),416(68.9),124(47.8),480(42.3),80(20.7),318(7.0),284(6.6)	N		S	
M108	14.69	sinapic acid sulfation+deoxidation+dihydroxylation isomer	$C_{11}H_{14}O_7S$	289.03874	289.03879	5.5	0.15	209(100.0),289(98.0),80(24.5),135(10.7),191(9.2),163(3.4),147(3.3),210(2.1)	N		S,C	
M109	14.70	6-[(methylsulfinyl)methyl]-1,3-thiazinan-2-thione deoxidation isomer	$C_6H_{11}NS_3$	194.01263	194.01257	1.5	-0.35	146(100.0),87(94.4),101(47.2),194(46.0),135(29.6),55(26.8),70(21.5),85(6.2)	P	S,C	S	
M110	14.90	sinapic acid isomer	$C_{11}H_{12}O_5$	223.06119	223.06079	6.5	-1.82	223(100.0),80(69.5),149(21.0),164(12.6),208(11.2),193(8.5)	N			S

M111	15.26	sinapine glucuronidation+deoxidation+dihydroxylation isomer	$C_{22}H_{34}NO_{10}$	472.21772	472.21759	6.5	-0.28	165(100.0),99(20.2),279(17.2),472(13.2),261(9.7),70(7.1),137(5.0)	P		S,C	
M112	15.48	sinapine methylation isomer	$C_{17}H_{26}NO_5$	324.18054	324.18024	5.5	-0.95	265(100.0),221(17.3),324(9.9),190(7.6),266(7.3),219(6.1),193(5.0),178(4.3),165(2.7),175(2.6)	P			C
M113	16.00	raphasatin dihydroxylation isomer	$C_6H_{11}NS_2$	162.04056	162.04053	1.5	-0.23	114(100.0),162(43.9),97(13.5),81(7.9),87(3.9),55(2.9),73(1.4)	P			C
M114	16.26	3,6'-disinapoylsucrose	$C_{34}H_{42}O_{19}$	753.22475	753.22498	14.5	0.30	205(100.0),190(78.2),175(32.2),223(12.4),547(12.2),149(9.4),753(6.1)	N	C	C	S,C
M115	16.57	3′-O-sinapoyl-6-O-feruloylsucrose isomer	$C_{33}H_{40}O_{18}$	723.21418	723.21466	14.5	0.65	175(100.0),160(93.6),205(77.9),190(68.7),723(15.2),517(12.1),547(10.4)	N			S,C
M116	16.82	helonioside A isomer	$C_{32}H_{38}O_{17}$	693.20362	693.20416	14.5	0.78	175(100.0),160(79.1),517(14.4),193(14.2),693(13.5),499(7.2)	N			S
M117	16.96	sinapic acid dihydroxylation isomer	$C_{11}H_{14}O_5$	225.07684	225.0764	5.5	-1.99	225(100.0),137(55.8),83(36.0),69(25.7),181(22.4),151(16.5),59(14.1),207(10.6),121(7.9)	N		S,C	
M118	17.34	glucoraphenin hydroxymethylation+glycination isomer	$C_{15}H_{26}N_2O_{12}S_3$	521.0575	521.05835	4.5	1.61	206(100.0),252(62.8),521(60.5),124(28.4),80(15.5),317(10.0),68(4.4)	N		S,C	
M119	17.510	6-sinapoylglucoraphenin isomer	$C_{23}H_{31}NO_{13}S_3$	624.08847	624.08868	9.5	0.33	97(100.0),96(51.5),624(35.4),149(17.9),80(15.8),223(15.7)	N			S,C

M120	18.96	Sinapic acid methylation+dihydroxylation isomer	$C_{12}H_{16}O_5$	239.09249	239.09221	5.5	-1.20	195(100.0),239(31.8),177(12.1),59(7.8),83(2.1),123(1.5),71(1.1),221(1.1)	N	S,C
M121	19.00	sinapic acid methylation isomer	$C_{12}H_{14}O_5$	237.07684	237.07655	6.5	-1.25	193(100.0),59(24.4),149(17.7),147(17.4),95(16.4),237(15.2),57(8.9),69(6.7),175(6.6)	N	S,C
M122	19.10	sinapic acid methylation+dihydroxylation isomer	$C_{12}H_{16}O_5$	239.09249	239.09222	5.5	-1.16	239(100.0),195(46.6),151(22.2),83(20.3),59(12.1),177(7.4),221(7.4),135(5.9),123(4.3)	N	S,C
M123	19.20	glucoraphenin hydroxymethylation isomer	$C_{13}H_{23}NO_{11}S_3$	464.03604	464.03693	3.5	1.91	206(100.0),252(82.2),464(80.3),124(31.6),80(17.8),204(4.2),85(3.4),207(3.0)	N	S,C

Note: S: raw RS; C: stir-fried RS; X: plasma; N: urine; F: feces; G: liver tissue

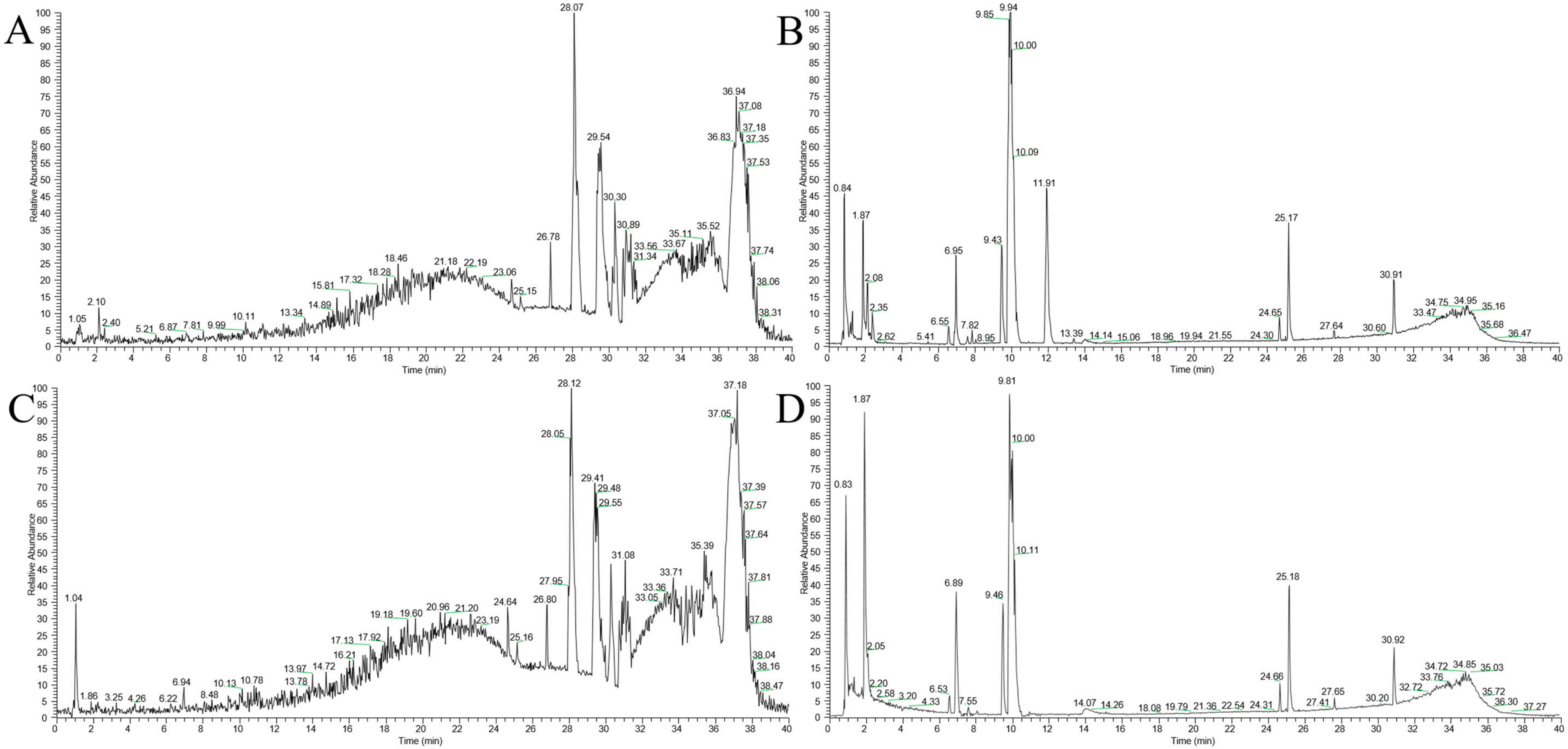

Figure S1. The chromatogram of raw and stir-fried RS decoctions (A: negative ion mode of raw RS decoction, B: positive ion mode of raw RS decoction, C: negative ion mode of stir-fried RS decoction, D: positive ion mode of stir-fried RS decoction).

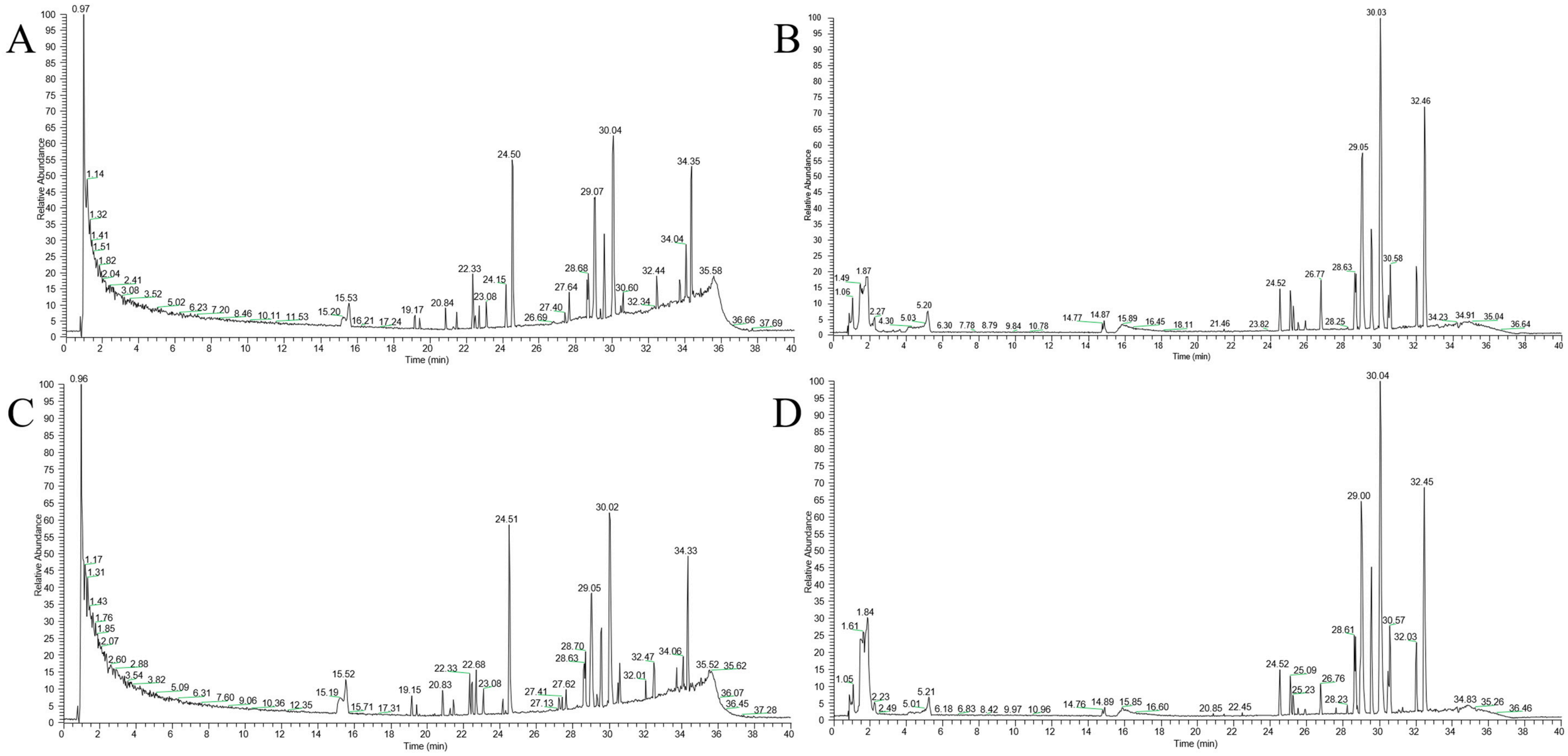

Figure S2. The chromatogram of raw and stir-fried RS decoctions in plasma (A: negative ion mode of raw RS decoction, B: positive ion mode of raw RS decoction, C: negative ion mode of stir-fried RS decoction, D: positive ion mode of stir-fried RS decoction).

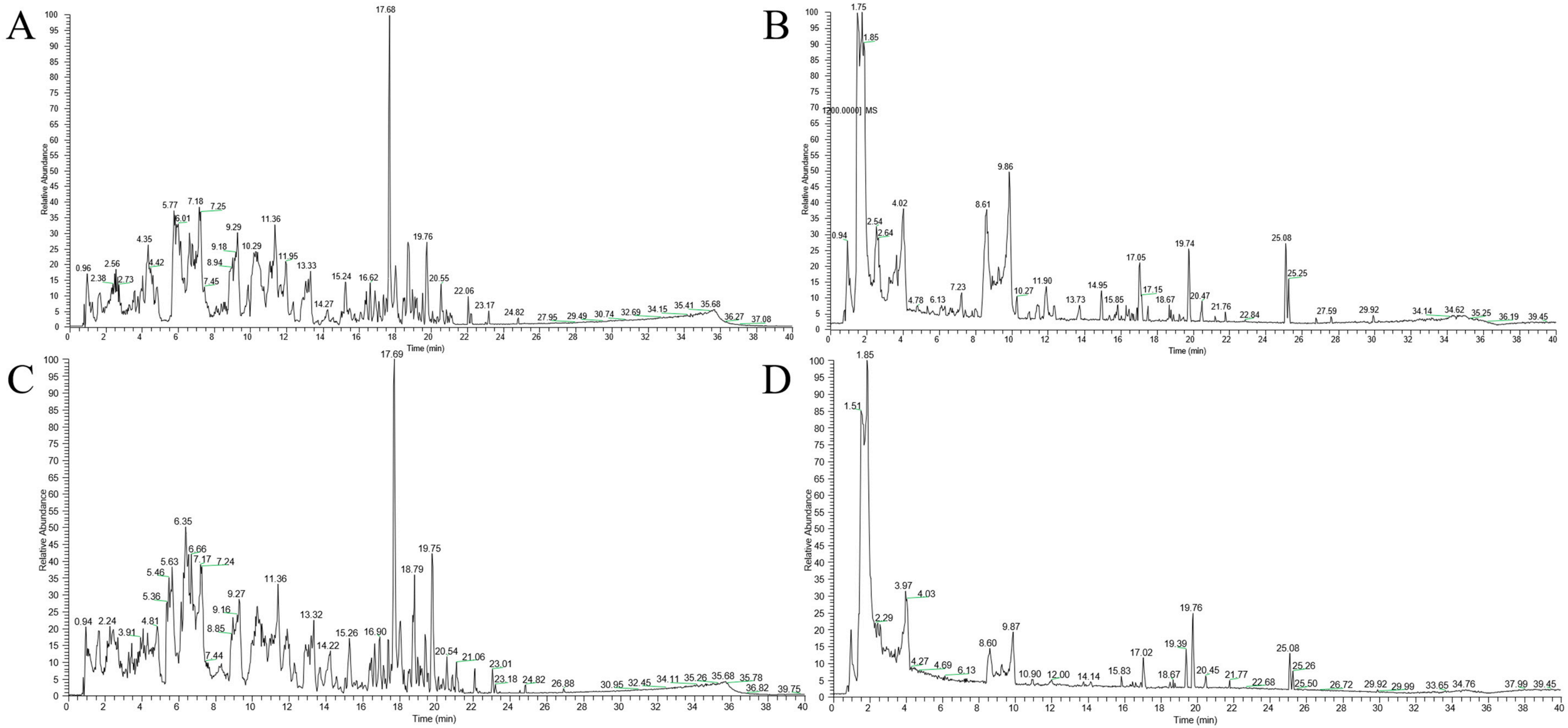

Figure S3. The chromatogram of raw and stir-fried RS decoctions in urine (A: negative ion mode of raw RS decoction, B: positive ion mode of raw RS decoction, C: negative ion mode of stir-fried RS decoction, D: positive ion mode of stir-fried RS decoction).

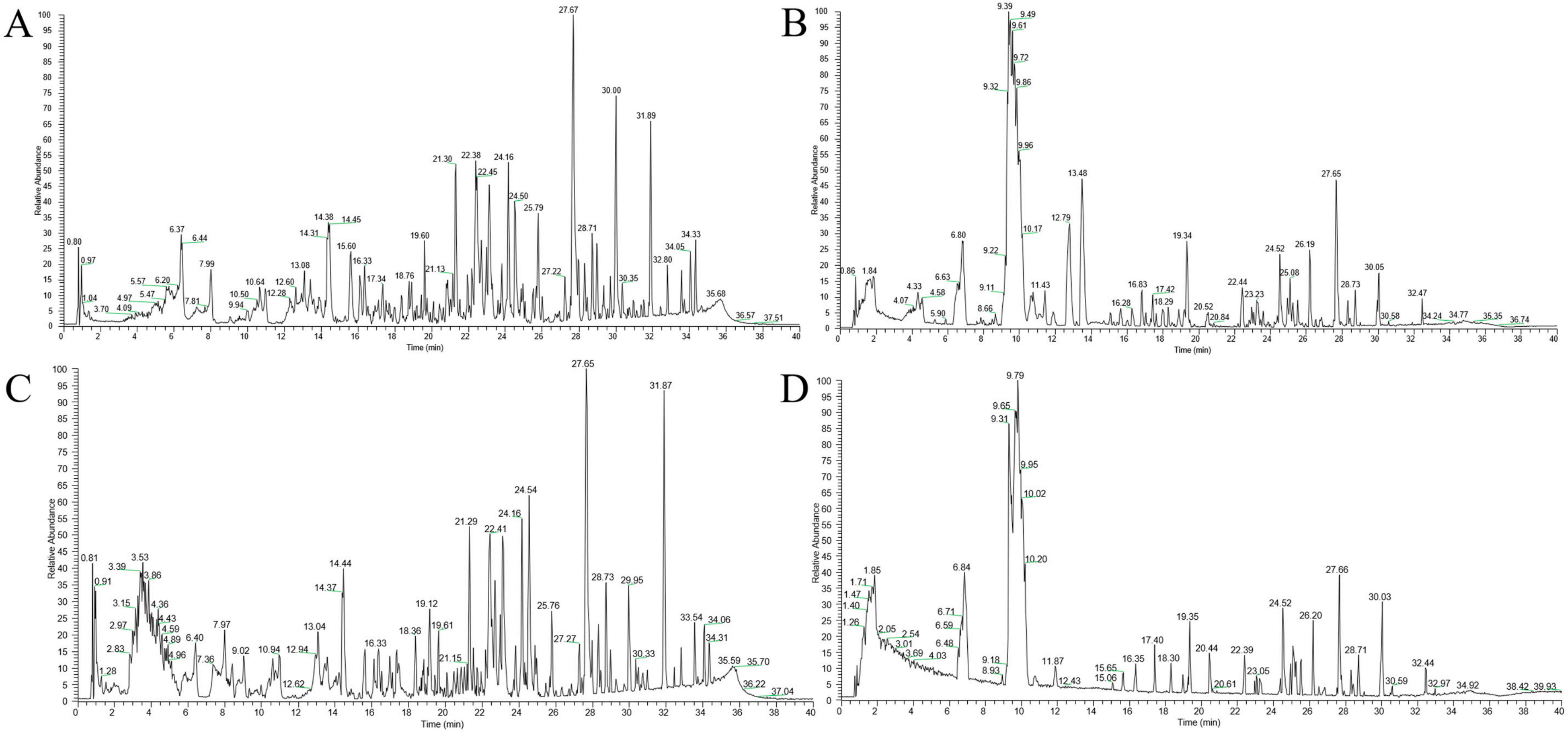

Figure S4. The chromatogram of raw and stir-fried RS decoctions in feces (A: negative ion mode of raw RS decoction, B: positive ion mode of raw RS decoction, C: negative ion mode of stir-fried RS decoction, D: positive ion mode of stir-fried RS decoction).

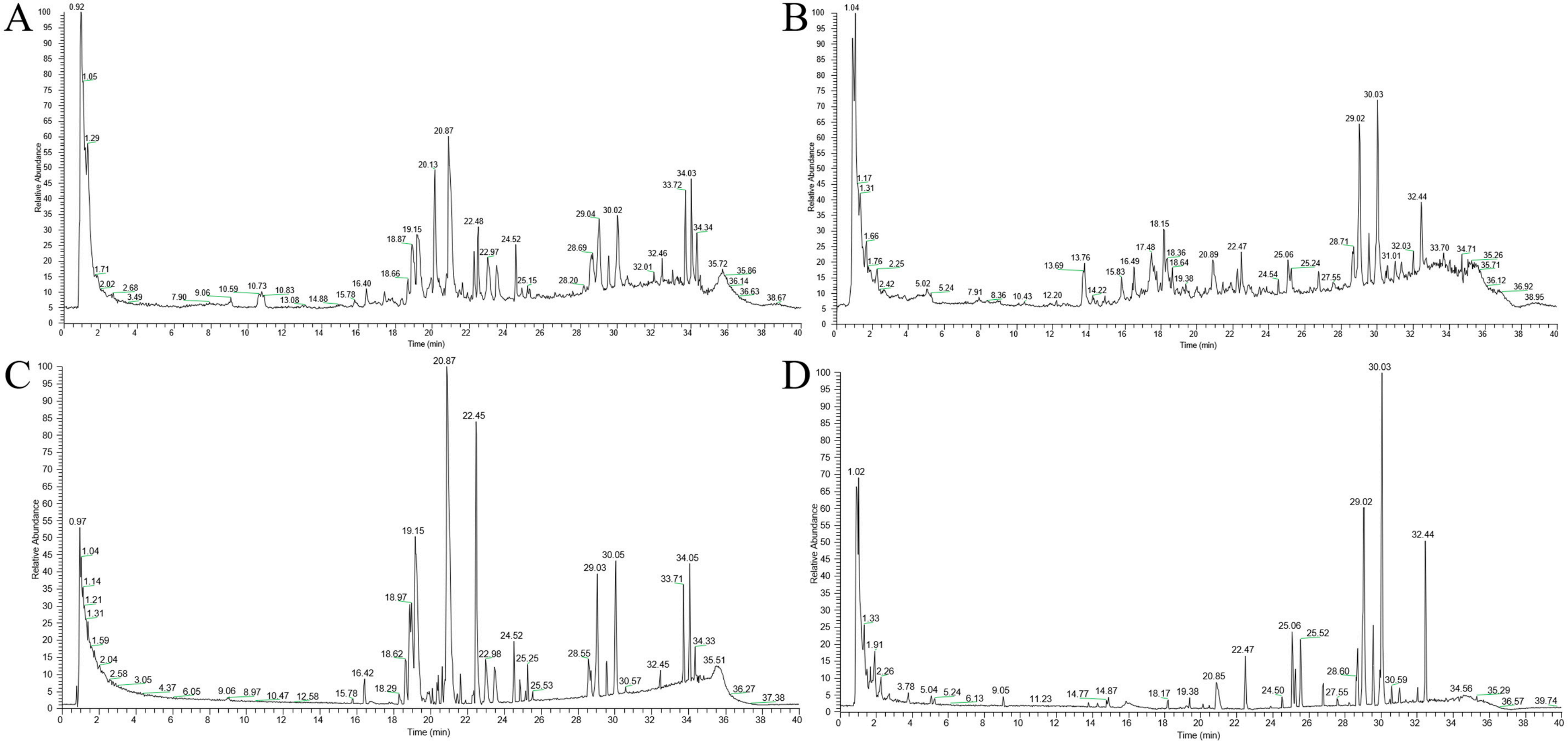

Figure S5. The chromatogram of raw and stir-fried RS decoctions in liver tissue (A: negative ion mode of raw RS decoction, B: positive ion mode of raw RS decoction, C: negative ion mode of stir-fried RS decoction, D: positive ion mode of stir-fried RS decoction).
